# Ascertainment bias from imputation methods evaluation in wheat

**DOI:** 10.1186/s12864-016-3120-5

**Published:** 2016-10-04

**Authors:** Sofía P. Brandariz, Agustín González Reymúndez, Bettina Lado, Marcos Malosetti, Antonio Augusto Franco Garcia, Martín Quincke, Jarislav von Zitzewitz, Marina Castro, Iván Matus, Alejandro del Pozo, Ariel J. Castro, Lucía Gutiérrez

**Affiliations:** 1 Statistics Department, Facultad de Agronomía, Universidad de la República, Garzón 780, Montevideo, 12900 Uruguay; 2 Biometris - Applied Statistics, Department of Plant Science, Wageningen University and Research Center, P.O. Box 16, 6700 AA Wageningen, Netherlands; 3 Departamento de Ciências Exatas, Escola Superior de Agricultura “Luiz de Queiroz” (ESALQ), Universidade de São Paulo (USP), CP 9, CEP 13400-970 Piracicaba, SP Brazil; 4 Programa Nacional de Investigación Cultivos de Secano, Instituto Nacional de investigación Agropecuaria, Est. Exp. La Estanzuela, Colonia, 70000 Uruguay; 5 Secobra Saatzucht GmbH, Feldkirchen 3, 85368 Moosburg, Germany; 6 Instituto de Investigaciones Agropecuarias, Centro Regional de Investigación Quilamapu, Casilla 426, Chillán, Chile; 7 Facultad de Ciencias Agrarias, Universidad de Talca, Casilla 747, Talca, Chile; 8 Department of Plant Production, Facultad de Agronomía, Universidad de la República, Ruta 3, Km.363, Paysandú, 60000 Uruguay; 9 Department of Agronomy, University of Wisconsin-Madison, 1575 Linden Dr, Madison, WI 53706 USA

**Keywords:** GBS, QTL, GWAS, Power, False positive

## Abstract

**Background:**

Whole-genome genotyping techniques like Genotyping-by-sequencing (GBS) are being used for genetic studies such as Genome-Wide Association (GWAS) and Genomewide Selection (GS), where different strategies for imputation have been developed. Nevertheless, imputation error may lead to poor performance (i.e. smaller power or higher false positive rate) when complete data is not required as it is for GWAS, and each marker is taken at a time. The aim of this study was to compare the performance of GWAS analysis for Quantitative Trait Loci (QTL) of major and minor effect using different imputation methods when no reference panel is available in a wheat GBS panel.

**Results:**

In this study, we compared the power and false positive rate of dissecting quantitative traits for imputed and not-imputed marker score matrices in: (1) a complete molecular marker barley panel array, and (2) a GBS wheat panel with missing data. We found that there is an ascertainment bias in imputation method comparisons. Simulating over a complete matrix and creating missing data at random proved that imputation methods have a poorer performance. Furthermore, we found that when QTL were simulated with imputed data, the imputation methods performed better than the not-imputed ones. On the other hand, when QTL were simulated with not-imputed data, the not-imputed method and one of the imputation methods performed better for dissecting quantitative traits. Moreover, larger differences between imputation methods were detected for QTL of major effect than QTL of minor effect. We also compared the different marker score matrices for GWAS analysis in a real wheat phenotype dataset, and we found minimal differences indicating that imputation did not improve the GWAS performance when a reference panel was not available.

**Conclusions:**

Poorer performance was found in GWAS analysis when an imputed marker score matrix was used, no reference panel is available, in a wheat GBS panel.

**Electronic supplementary material:**

The online version of this article (doi:10.1186/s12864-016-3120-5) contains supplementary material, which is available to authorized users.

## Background

Genetic markers are nowadays an essential part of plant and animal breeding programs. Next-generation sequencing (NGS) techniques allow discovering, sequencing, and genotyping thousands of Single Nucleotide Polymorphism (SNPs) covering the whole genome [1]. These SNPs are being used in analyses like transcriptome assembly [2], generation of high-quality draft genomes even for complex genomes [3], understanding plant growth [4], evaluating the effect of epigenetics in plant development [5], isolation of mutant genes [6],species evolution and economic insight [7], genetic diversity [8], GWAS [9], and GS [10]. The GBS technique is one of the most used NGS approaches [8–11]. It was developed originally for barley and maize, and later extended to other complex genomes species like wheat [8–11]. GBS that relies on methylation-sensitive restriction enzymes is highly efficient [12]. However, GBS generates a large proportion of missing data when alleles are obtained due to the use of short reads and when low sequencing depth are used [12]. Therefore, different strategies to impute missing data have been developed and used for genetic analyses [9]. Some imputation methods use reference panels and are based on Linkage Disequilibrium (LD), while other methods do not require reference panels. In the first group, the most common methods are known as MACH [13], IMPUTE [14], fastPHASE [15], PLINK [16], and Beagle [17]. All of them use haplotype segments from a reference panel densely genotyped to impute missing markers [18–20]. MACH uses a Markov Chain based algorithm to infer pairs of haplotypes for each individual’s genotypes [13]. IMPUTE considers the sequence of pairs of known haplotypes as hidden states, then models the sequence of hidden states based on a recombination map estimated from the reference data, and finally it predicts unknown genotypes [14]. The fastPHASE algorithm is a haplotype clustering algorithm that samples missing genotypes based on allele frequencies estimated from reference haplotypes, and then uses an Expectation- Maximization (EM) algorithm to estimate parameter values to infer missing genotypes [15]. PLINK predicts missing data by the local haplotypic background and by the haplotype formed by the two or more flanking SNPs [16]. Finally, Beagle is a haplotype clustering based algorithm that uses the localized haplotype cluster model to group haplotypes at each marker and then finds the most likely haplotype pairs based on the individual’s known genotypes [17]. Therefore, strong LD among markers and low minor allele frequency (MAF) is required for effective LD imputation methods [21]. Additionally, more markers with an even genome coverage and therefore smaller distance among markers, and markers with larger subpopulation differentiation are also desirable to ensure imputation accuracy [22]. The second group of methods do not require a reference panel and include imputation by the mean, the MVN-EM algorithm, and random forests [10]. In mean imputation, the most common allele at a particular marker in the population is used to impute missing data. MVN-EM, on the other hand, considers the realized additive relationship matrix between the lines and an EM approach assuming that marker genotypes follow a multivariate normal distribution designed for use with GBS. Finally, random forest methods use an algorithm with multiple decision trees to determine a prediction value for each missing data point. For an overview of the imputation methods see [10].

Several studies found that imputation can improve QTL power detection [23, 24], but other studies found that large power is accompanied by either larger false positive rates or an increase in the multiple-testing penalty [20, 25]. Unless a ‘one-hit’ procedure is used (i.e. the uncertainty of genotypic probability distributions due to the imputation is incorporated in the GWAS analysis), large imputation error can be generated [26]. Other studies found that imputation should be carefully evaluated because quality control of the data is an important source of loss of power [27]. To carry on GWAS analysis, where one marker at a time is being tested, marker-trait associations can be estimated without marker imputation using the available information at each marker.

The aim of this study was to compare the performance of imputation methods for GWAS analysis when no reference panel is available in a wheat GBS panel. Specifically, our objectives were: (1) to evaluate the effect of imputation using a golden standard (i.e. simulation over a complete marker score matrix), to determine whether ascertainment bias is responsible for imputation success; (2) to evaluate whether the outcome of the imputation performance is affected by the marker score matrix used to simulate the QTL; and (3) to compare the effect of imputation in a real phenotype wheat panel using GBS data with different missing rates (25 %, 35 % and 50 %) and four phenotypic traits.

## Results

**Fig. 1:**
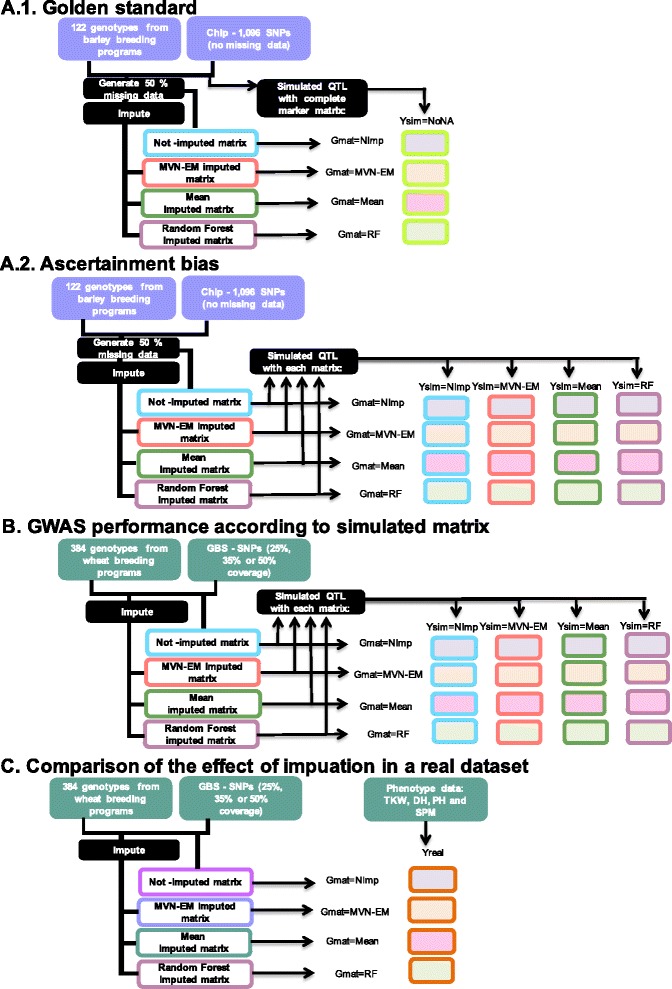
General scheme of the procedures we followed for each component. **a** Procedures for golden standard (A.1) and ascertainment bias (A.2); **b** Procedure for GWAS performance based on simulated matrix; **c** Procedure for comparison of the effect of imputation in a real phenotypic dataset. Each procedure details the germplasm, genotypic and phenotypic dataset used, as well as simulation approach to obtain each phenotypic vector and GWAS analysis marker score matrices used. Procedures that used wheat data are in green and procedures that used barley data are in purple. DH, Days to Heading; GBS, Genotype-by-sequencing; MVN-EM, Multivariate Normal Expectation Maximization; Not-imputed marker score matrix; NoNA, No missing data marker score matrix; PH, Plant Height; QTL, Quantitative Trait Loci; RF: Random Forest marker score matrix; SNPs, Single-Nucleotide Polymorphism; SPM, Spikes Per Square Meter; TKW, Thousands Kernel Weight

The strategies we pursued are explained in the Methods section, and the general procedure presented in Fig. 1. We used different number of QTL and heritabilities to simulate the QTL, along with different thresholds for calling the QTL. We summarized the results with power (*PO)* and false positive rate (*FPR)*.

### Ascertainment bias in imputation performance comparison (golden standard)

**Fig. 2:**
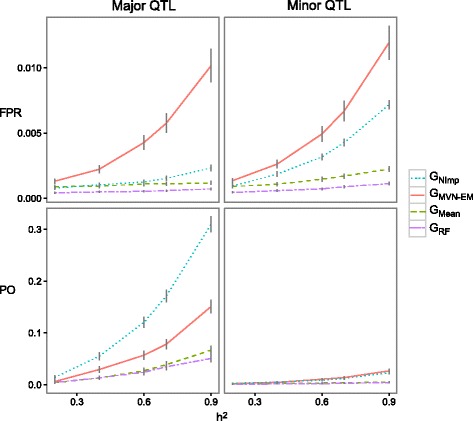
Power (*PO*) and false positives rate (*FPR*) for major and minor QTL with 25 QTL, for the golden standard from barley with a Bonferroni threshold corrected by the effective number of independent markers. Each parameter was calculated for the combinations of: heritabilties (*h* ^*2*^), a marker score matrix to simulate the QTL (i.e. Y_sim-*NoNA*_), and marker score matrices to perform the GWAS analysis (i.e. G_*NImp,*_ G_*MVN-EM,*_ G_*Mean*_ and G_*RF*_)

When we used a golden standard matrix of barley for simulating the QTL (i.e. a complete dataset, for general approach see Fig. 1A1), we found that for major QTL effects, larger power was obtained without imputing the genotypic matrix. Furthermore, for minor QTL effects, larger power was detected without imputing the genotypic matrix or imputing it with the MVN-EM method (G_*NImp*_, G_*MVN-EM*_ Fig. 2). The smallest false positive rate was obtained for the genotypic matrix imputed by the RF method (G_*RF*_), and the largest false positive rate was obtained with the *MVN-EM* imputation method (G_*MVN-EM*_). False positive rates were still really small (i.e. 0.015, Fig. 2). Power was also small in general (i.e. 0.3, Fig. 2). The same pattern was found when using different threshold levels for the dissection of quantitative traits (i.e. Bonferroni corrected by the effective number of independent markers, Fig. 2; Bonferroni correction, Additional file 1; and an arbitrary threshold set at α = *0.01*, Additional file 2).

**Fig. 3:**
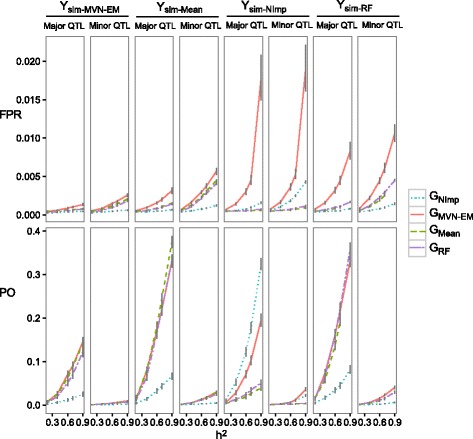
Power (*PO*) and false positives rate (*FPR*) with 25 QTL, for major and minor QTL for ascertainment bias in imputation performance comparison in barley, with a Bonferroni threshold corrected by the effective number of independent markers. Each parameter was calculated for the combinations of: heritabilties (*h* ^*2*^), marker score matrices to simulate the QTL (i.e. Y_sim-*NImp*_, Y_sim-*MVN-EM,*_ Y_sim-*Mean*_ and Y_sim-*RF*_), and marker score matrices to perform the GWAS analysis (i.e. G_*NImp,*_ G_*MVN-EM,*_ G_*Mean*_ and G_*RF*_)

When we simulated QTL over an imputed matrix (for the general approach see Fig. 1A.2), we found that larger power was obtained with the imputed genotypic matrices (G_*Mean,*_ G_*MVN-EM*_ or G_*RF*_), while the largest false positive rate was obtained with the *MVN-EM* imputation method (G_*MVN-EM*_) (Fig. 3). However, when QTL were simulated over a not-imputed matrix, the largest power was obtained when a not-imputed or imputed by the MVN-EM genotypic matrices were used (G_*MVN-EM*_ or G_*NImp*_). This pattern was consistent across number of QTL (i.e. 25 and 50, data not shown) and heritabilities (i.e. 0.2, 0.4, 0.6, 0.7, 0.9, Fig. 3). The same pattern was found when using different threshold levels for the dissection of quantitative traits (i.e. Bonferroni corrected by the effective number of independent markers, Fig. 3; Bonferroni correction, Additional file 3; and an arbitrary threshold set at α = *0.01*, Additional file 4).

### Imputation effect for real GBS data with 25 %, 35 % or 50 % missing information

**Fig. 4:**
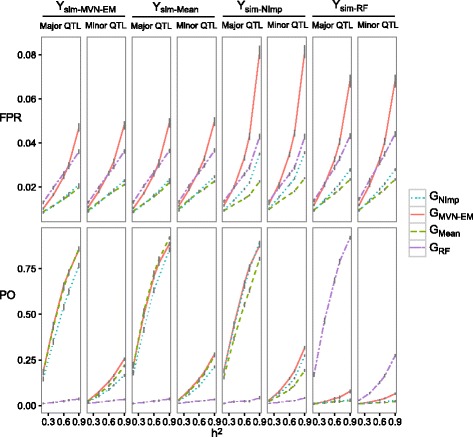
Power (*PO*) and false positives rate (*FPR*) with 25 QTL and 50 % missing rate, for major and minor QTL to evaluate the GWAS performance based on simulated matrix with a Bonferroni threshold corrected by the effective number of independent markers. Each parameter was calculated for the combinations of: heritabilties (*h* ^*2*^), marker score matrices to simulate the QTL (i.e. Y_sim-*NImp*_, Y_sim-*MVN-EM,*_ Y_sim-*Mean*_ and Y_sim-*RF*_), and marker score matrices to perform the GWAS analysis (i.e. G_*NImp,*_ G_*MVN-EM,*_ G_*Mean*_ and G_*RF*_)

By using naturally sparse genotypic matrices like GBS in wheat with 25 %, 35 % or 50 % missing data information (for the general approach see Fig. 1b), we detected that larger power was obtained when a not-imputed or imputed by the MVN-EM genotypic matrices were used (Fig. 4, Additional files 5 and 6). However, when simulating over a matrix with imputed data, larger power was obtained by recover QTL with an imputed matrix (Fig. 4, Additional files 5 and 6). This was true for the different number of QTL (i.e. 25 and 50, data not shown) and heritabilities (i.e. 0.2, 0.4, 0.6, 0.7, 0.9, Fig. 4, Additional files 5 and 6). Differences between power were more evident for major QTL, resulting in a reasonable increase of power for high heritabilities (Fig. 4). The largest values of false positive rate were found when simulating with the Y_sim-*NImp*_ and G_*MVN-EM*_ or the Y_sim-*RF*_ and G_*MVN-EM*_ (Fig. 4, Additional files 5 and 6). Additionally, the same pattern was found using different threshold levels (i.e. Bonferroni corrected by the effective number of independent markers, Fig. 4; Bonferroni correction, Additional file 7; and an arbitrary threshold set at α = *0.01*, Additional file 8).

### Imputation effect on GWAS for real phenotypes

**Fig. 5:**
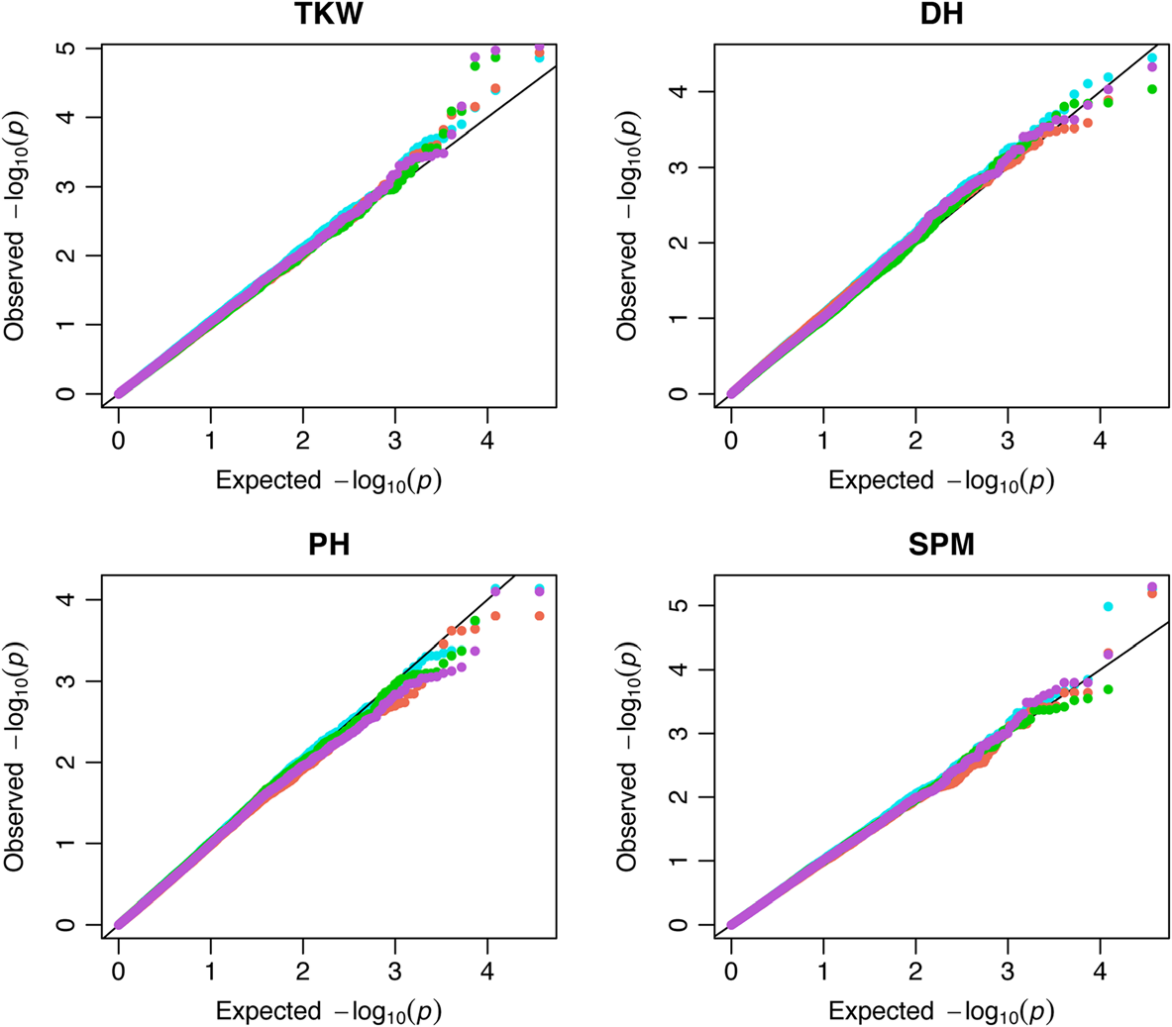
QQ plots of the p-values resulted from the GWAS analysis from real phenotype wheat data with 50 % missing rate and a Bonferroni threshold corrected by the effective number of independent markers. For each trait measured and each marker score matrix evaluated, a qq-plot of the *p*-values from the GWAS analysis is presented. The marker score matrices were: *NImp* (not imputed) in turquoise*, Mean* (mean imputed) in green, *MVN-EM* (Multivariate Normal Expectation Maximization method) in coral and *RF* (Random Forest method) in orchid. The phenotype traits are: DH, days to heading; PH, Plant Height; SPM, Spikes Per Square Meter; TKW, Thousands Kernel Weight

**Fig. 6:**
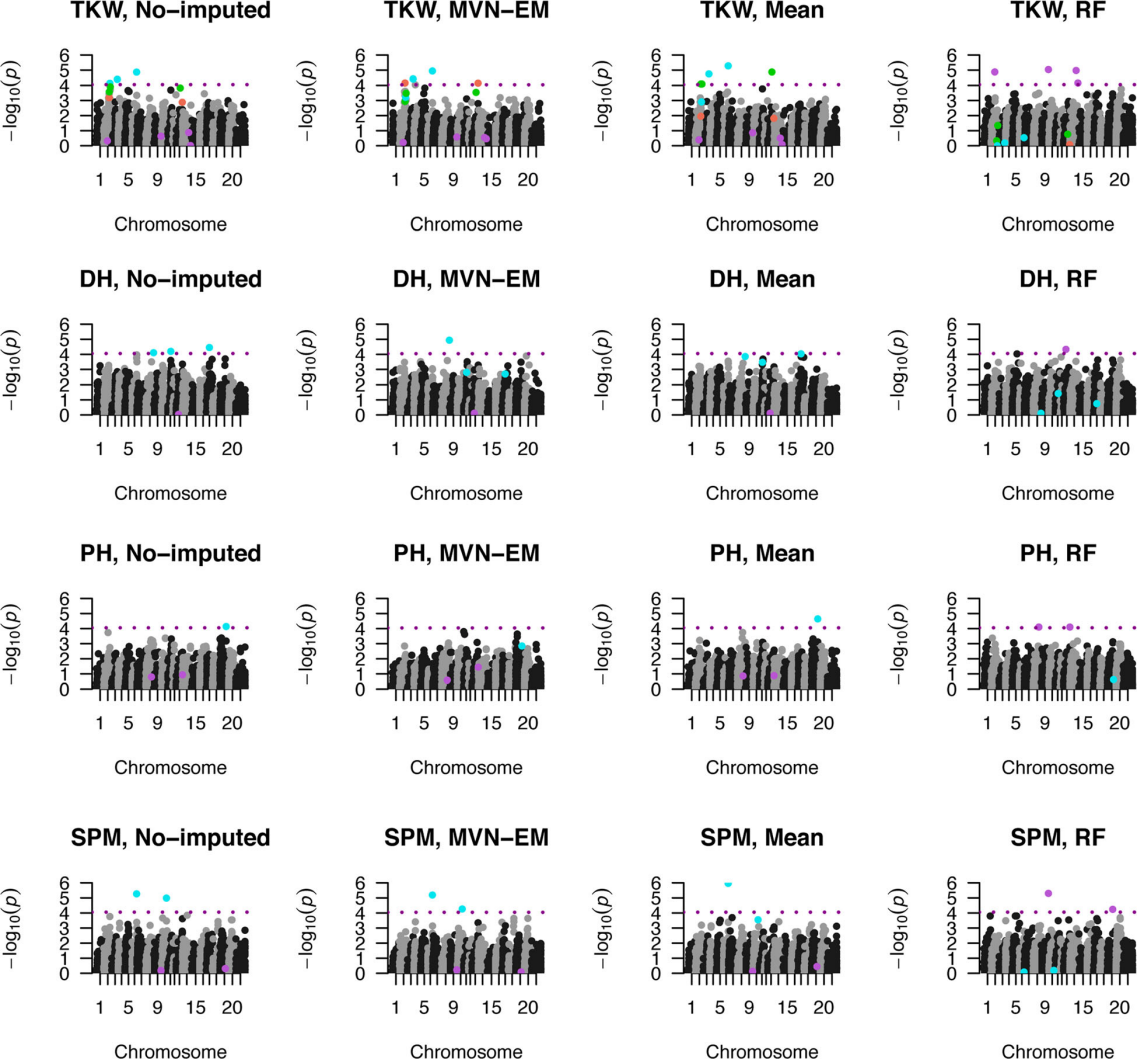
Manhattan plots of the GWAS analysis for real phenotype wheat data with 50 % missing rate and a Bonferroni threshold corrected by the effective number of independent markers. For each trait measured and each marker score matrix evaluated, a manhattan plot of the GWAS analysis is presented. The phenotypic traits are: DH, Days to Heading; PH, Plant Height; SPM, Spikes Per Square Meter; TKW, Thousands Kernel Weight. The marker score matrices were: *NImp* (not imputed)*, Mean* (mean imputed), *MVN-EM* (Multivariate Normal Expectation Maximization method) and *RF* (Random Forest method). QTL detected by the *NImp* matrix are in turquoise, QTL detected exclusively by the *MVN-EM* matrix are in coral, QTL detected exclusively by the *Mean* matrix are in green, and QTL detected exclusively by the *RF* matrix are in orchid

We compared the QTL obtained for GWAS analysis using real phenotypic data from wheat, between the not-imputed matrix (G_*NImp*_) with different missing rates (25 %, 35 % and 50 % of missing data), and the genotypic data imputed with the mean, MVN-EM or RF method (G_*Mean*_, G_*MVN-EM*_ or G_*RF*_). The performance of GWAS analysis was similar across imputation methods (Fig. 5, Additional files 9 and 10), but not all QTL were detected across methods. For the 4 traits, plant height (PH, cm), days to heading (DH, days), thousand kernel weight (TKW, g) and spikes per square meter (SPM, number, Fig. 6, Additional files 11 and 12), we detected different putative QTL when using imputed or not-imputed matrices. In general, the *MVN-EM* imputation method performed similarly to non imputation, having some QTL being detected by both methods (Fig. 6, Additional files 11 and 12). However, each approach found also unique QTL (Fig. 6, Additional files 11 and 12).

### Differences between methods for false positive rate

When we performed FPR boxplots with the replications for analyzing if the differences between the methods are significantly different or due to random errors (Additional files 13, 14, 15, 16, 17), we found that FPR rates were larger for: (i) the imputed genotypic matrices by the MVN-EM method for the golden standard, (ii) the imputed genotypic matrix by the MVN-EM method (G_*MVN-EM*_) for the ascertainment bias, (iii) the imputed genotypic matrices by the MVN-EM or RF methods (G_*MVN-EM,*_ G_*RF*_) for the GBS data with 35 % or 50 % missing data, (iv) and the imputed genotypic matrices by the RF method (G_*RF*_) for the GBS data with 25 % missing data.

## Discussion

New whole-genome genotyping techniques are constantly being developed and used for genetic analyses like GWAS [9]. Although GBS is a powerful tool for genotyping hundreds of individuals with thousands of SNPs, it generates large amounts of missing information, and therefore, researchers have applied several strategies to impute these missing [14–17]. However, when retained a considerable amount of missing information using GBS data in wheat or artificially removing genotypic data from complete panels in barley, we found that imputation does not improve the dissection of quantitative traits performance in several situations. Our results should be restricted to our panels that have a specific LD (barley and wheat) and SNP quality, due to the continuous improvement of the sequencing technologies that allows the decrease of costs and therefore the increase of sequencing depth and quality, leading to a lower missing rate.

### Ascertainment bias in imputation performance comparison (golden standard)

When we used the “golden standard” marker score matrix, the not-imputed marker score matrix outperformed the imputation methods for all the combinations of parameters (Fig. 2, Additional files 1 and 2). The higher values of false positive rate found with the *MVN-EM* matrix and lower values of power found with the *RF* matrix for all thresholds could be a consequence of an imputation error affecting the signal of the QTL.

The fact that we also found that the not-imputed marker score matrix outperformed the imputation methods comparing both, power and false positive rate simultaneously, when we used real GBS data (i.e. data with missing points, Fig. 4), suggests that using an imputed matrix for GWAS analysis could introduce an ascertainment bias. This could be caused when there is no reference panel, and the uncertainty of genotypic probability distributions due to the imputation is not considered, as methods based on LD have found that if some restrictions are taken into account (i.e. strong LD among markers, low minor MAF, short distances between not-imputed markers, and markers with higher subpopulation differentiation), the imputation accuracy and then the GWAS is improved [22, 28].

Although the low power found to detect QTL for the barley marker score matrix could theoretically be due to low LD between markers in the same LD blocks, we do not expect this to be the reason of low power in our study. When there are unlinked QTL controlling a trait, the power is moderate even with large populations and high heritabilities [29]. However, we do not expect unlinked QTL within the LD blocks due to the cluster of markers within those blocks [30], and because the genome coverage of the markers was very high, having 50 % of its SNPs, at a distance smaller than 0.625 cM (Table 1). The small population (122 lines) used for barley dataset could be the reason affecting the low values of power detected, as the power is a function of the population size [31]. However, this should not differently affect the imputation methods. Additionally, the great differences found in power and false positive rate between major and minor QTL, could indicate that major QTL are the QTL mostly detected by any of the imputation methods. Other LD structures in different populations could make our results to vary, therefore, this results are restricted to the populations used in this analysis. Further analyses considering different population structure should be tested.

**Table 1:** SNPs coverage on the golden standard matrix (i.e. complete SNP array), indicating for each chromosome (Chr = chromosome), the number of SNPs, the length (in cM), the largest gap without markers (cM), the median distance between pairs of adjacent markers, and the 25 % and 75 % quantiles of the adjacent marker distances

Chr	SNPs number	Length (cM)	Largest gap (cM)	Median (cM)
1	125	139.78	10.74	0.63
2	187	150.27	8.21	0.58
3	178	170.88	6.59	0.58
4	131	121.65	7.50	0.60
5	201	194.03	8.05	0.57
6	147	129.38	8.62	0.47
7	127	166.56	10.53	0.49

### Imputation effect for real GBS data with 25 %, 35 % or 50 % missing information

The differences found when we simulated QTL on top of imputed or not-imputed marker score matrices (Fig. 4, Additional files 5 and 6) were probably due to the imputation method used and the simulation. Therefore, we found that not-imputing was the best option for evaluating one marker at a time in GWAS analysis using GBS data with 25 %, 35 % or 50 % missing information, especially for detecting major QTL.

### Imputation effect on GWAS for real phenotypes

As no significant differences were detected in the real wheat datasets in terms of the type 1 error inflation imputation (Fig. 5, Additional files 9 and 10), we consider that imputation does not improved the GWAS performance and therefore is not needed.

The traits evaluated in this paper were selected for having high heritability values and being related or a component of grain yield. The high heritability values may have reduced the differences between the QTL found with G_*NImp*_ or G_*MVN-EM*_.

We found QTL where previous QTL were reported. The QTL found for TKW (chromosome 1B, bin 224 and 242) with the G_*NImp*_, G_*MVN-EM*_ and G_*Mean*_ matrices for 50 % missing data, and with the G_*Mean*_ matrix for 25 % and 35 % missing data, are partially coincident with a QTL reported for green leaf area [32], a QTL reported for Near Differential Vegetative Index [33] and a QTL reported for yield, anthesis and plant height [34]. A QTL found for TKW (chromosome 1D, bin 205) with the G_*NImp*_, G_*MVN-EM*_ and G_*Mean*_ matrices for 25 %, 35 % and 50 % missing data, is coincident with a QTL reported for grain yield and plant height [34]. The QTL found for TKW (chromosome 2D, bin 167) with 3 marker score matrices (G_*NImp,*_ G_*MVN-EM*_ and G_*Mean*_) for 25 %, 35 % and 50 % missing data, SPM (chromosome 2D, bin 167) with the G_*NImp*_ matrix for 25 % and 50 % missing data, and with 3 marker score matrices (G_*NImp,*_ G_*MVN-EM*_ and G_*Mean*_) for 35 % missing data, are coincident with a QTL reported for kernel weight, Near Differential Vegetative Index and flag leaf [33]. A QTL found for DH (chromosome 3B, bin 282) with G_*NImp*_ *and* G_*MVN-EM*_ for 50 % missing data is coincident with a QTL reported for grain filling duration [32]. A QTL found for SPM (chromosome 4A, bin 179) with the G_*NImp*_ and G_*MVN-EM*_ matrices for 25 %, 35 % and 50 % missing data, is coincident with a QTL reported for anthesis and plant height [34]. The QTL found for DH (chromosome 4B, bin 106) with the G_*NImp*_ matrix for 50 % missing data, is coincident with a QTL reported for yield and plant height [34]. A QTL found for DH (chromosome 6B, bin 116) with the G_*NImp*_ matrix for 35 % and 50 % missing data, and with the G_*NImp*_ and G_*Mean*_ matrices for the 25 % missing data, is coincident with a QTL for yield and plant height [28]. A QTL found for PH (chromosome 7A, bin 225) with the G_*NImp*_ and G_*Mean*_ matrices for 50 % missing data, is coincident with yield and anthesis [34]. These positions are based on bins and should be regarded as an approximation. These could be improved after the draft of the genome is available [35].

As we found that QTL detected by the G_*NImp*_ and G_*MVN-EM*_ matrices were similar, we believe that imputation do not improve GWAS analysis.

## Conclusions

Imputation can introduce an ascertainment bias to GWAS analysis using GBS within crops when a reference panel is not available. Comparing the GWAS performance by the power and false positive rate with imputed or not-imputed marker score matrices, poorer performance was found when an imputed marker score matrix was used. Additionally, the power and false positive rate changed in a clear way between major and minor QTL, showing that differences among imputation methods were more evident for major QTL and that the detection of minor QTL is negligible. Our results are restricted to the wheat panel used, as with different LD they could vary, and as well with different GBS quality data, which is affected by different SNP identification algorithms.

## Methods

### Dataset

We used two datasets: (1) a complete SNPs barley panel array (i.e. 99 % coverage), and (2) a GBS wheat marker score matrix with an average of 25 %, 35 % or 50 % missing points and phenotypic data (for general approach see Fig. 1).

The complete barley SNP marker score array dataset (Additional file 18), consisted in a panel of 122 barley advanced inbred lines from a population of 360 described in [36]. Briefly, 1,096 SNPs from the Barley Oligonucleotide Pool Assay-1 (BOPA 1, Additional file 19) were selected [37, 38]. A total of 122 lines were chosen to form 2 complete datasets without missing information (Table 1).

The wheat GBS dataset (Additional file 20), consisted on a panel of 384 advanced inbred lines from breeding programs: 186 genotypes from the National Wheat Breeding Program from Uruguay (INIA-Uruguay, National Institute of Agricultural Research), 55 genotypes from the National Wheat Breeding Program from Chile (INIA-Chile), and 143 genotypes from the International Breeding Center of Maize and Wheat (CIMMYT, International Maize and Wheat Improvement Center), published in [39]. The CIMMYT genotypes share common ancestors with the INIA-Chile genotypes (see [39] for more details). DNA was extracted by the DNeasy Plant Maxi Kit (QIAGEN). Library construction was conducted at Kansas State University (Manhattan, Kansas) using a PstI-MspI GBS protocol [10]. The sequencing was performed on an Illumina Hi-Sequation 2000 at the DNA core facility at the University of Missouri, Columbia, Missouri, and the McGill Univesity-Génome Quebec Innovation Centre (Montreal, Canada) for each set of libraries. SNPs were obtained using the Tassel-GBS Pipeline [40]. The base quality and distribution of sequences was studied with the Galaxy (https://galaxyproject.org/) software. SNPs with more than 25 %, 35 % or 50 % missing points and with minor allele frequency (MAF) smaller than 10 % were excluded. Sequences were blasted to the SyntheticxOpata map (synop) using the blastn function from NCBI-BLAST+ package using the number of descriptions and the number of threads set to one. Therefore, SNPs were placed into recombination bins [11] (Additional file 21). A final matrix set of 18,337 SNPs was obtained for 50 % missing data (Table 2), a final matrix set of 8,227 SNPs was obtained for 25 % missing data (Additional files 22 and 23), and a final matrix set of 11,858 SNPs was obtained for 35 % missing data (Additional files 24 and 25).

**Table 2:** SNPs coverage on the GBS genotypic matrix with 50 % coverage, indicating for each chromosome (Chr = chromosome), the number of SNPs, the length (in cM) and the largest gap without markers (cM)

Chr	SNPs number	Length (cM)	Largest gap (cM)	Median (cM)
1A	821	266	33	0
1B	1282	294	22	0
1D	255	242	25	0
2A	900	242	22	0
2B	1746	266	38	0
2D	327	182	27	0
3A	929	329	28	0
3B	1912	290	30	0
3D	270	287	29	0
4A	907	234	28	0
4B	610	177	31	0
4D	74	130	45	0
5A	1023	232	26	0
5B	1270	316	22	0
5D	197	306	29	0
6A	883	237	34	0
6B	1302	232	25	0
6D	243	276	28	0
7A	1456	323	24	0
7B	1660	263	40	0
7D	270	337	45	0

The phenotypic data for the wheat panel was obtained from an evaluation in a Mediterranean environment in Santa Rosa-Chile in 2011 (36° 329’ S, 71° 559’ W; 217 m.a.s.l.). The field was irrigated with 50 mm m^-2^ at each of four moments: tillering, flag leaf emergence, heading date, and grain filling (see [33] for further details). The experimental design was an alpha-lattice with 20 replications and 20 incomplete blocks. The traits evaluated were: plant height (PH, cm) evaluated from the base of the plant to the flower insertion (Additional file 26), days to heading (DH, days) was recorded when 50 % of the culms showed emerged ears (Additional file 27), thousands kernel weight (TKW, g, Additional file 28), and spikes per square meter (SPM, number, Additional file 29). We obtained the best linear unbiased predictors (BLUPs) for each genotype using the following model for each trait: *y*_*ijk*_ = *µ* + *a*_*i*_ + *β*_*j*_ + *δ*_*k*(*j*)_ + *ε*_*ijk*_ where *y*_*ijk*_ is the value for the phenotypic trait corresponding to the *i-th* genotype, *j-th* replication, and *k-th* incomplete block, μ is the overall mean, *a*_*i*_ is the random effect of the *i-th* genotype with *a*_*i*_ ~ N(0, *σ*_*g*_^2^), *β*_*j*_ is the effect of the *j-th* replication, *δ*_*k*(*j*)_ is the random effect of the *k-th* incomplete block within the *j-th* replication with *δ*_*k*(*j*)_ ~ N(0, *σ*_*B*_^2^), *ε*_*ijk*_ is the experimental error corresponding to the *i-th* genotype, *j-th* replication and *k-th* incomplete block with *ε*_*ijk*_ ~ N(0, *σ*_*e*_^2^). The genotypic breeding values were estimated with the function *lmer* (*lme4* package) in R statistical software [41]. Broad sense heritabilities were estimated in R statistical software [35] using the above model (Table 3).

**Table 3:** Broad sense heritability (*h* ^*2*^) for the real wheat panel for all traits in Santa Rosa- Chile 2011

Trait	Santa Rosa- Chile 2011
Plant height (cm)	0.78
Days to heading (days)	0.97
Thousand kernel weight (g)	0.93
Spikes per square meter (number)	0.76

### Imputation methods

For the barley SNP array panel, we started with a genotype by marker score matrix with 122 genotypes (rows) and 1,096 markers (columns) without missing values Markers were scored as {1, -1}. Then, we randomly generated missing values in order to have the same coverage as the GBS panel (50 %). Finally, three methods were used to fill in those missing values, MVN-EM, which considers the realized additive relationship matrix between the lines and an EM approach assuming that marker genotypes follow a multivariate normal distribution [10], Random Forest (RF), which uses an algorithm with multiple decision trees to predict a value for each missing point, and the Mean, which uses the average value score per marker (i.e. the expected allele value at the particular marker). Imputation was conducted in R statistical software [41] with the *A.mat* function (*rrBLUP* package) [42].

For the wheat GBS panel, we started with: (i) a genotype by marker score matrix with 384 genotypes (rows) and 18,337 markers (columns) with 50 % of missing values, (ii) a genotype by marker score matrix with 384 genotypes (rows) and 8,227 markers (columns) with 25 % of missing values, and (iii) a genotype by marker score matrix with 384 genotypes (rows) and 11,858 markers (columns) with 35 % of missing values. Markers were scored as the number of alleles {NA, 1, -1}. We used the same methods as the previous sections to impute by the MVN-EM and the Mean.

### Simulation procedure

To evaluate the effect of imputation using a golden standard with the barley SNP array, we created phenotypic vectors simulating QTL on top of the complete barley marker score matrix (Y_sim-*NoNA*_). The phenotypic vectors were the sum of the effects of genotypic and residual terms, Y_sim_ = g + e. The genotypic effect was calculated as the sum of the markers (selected as QTL) effects and markers effects were obtained from a Beta(2, 6) distribution. Markers selected as QTL were obtained from the LD blocks defined from a single linkage agglomerative procedure [30] with euclidean distances between markers and a minimum of 1.5 cM to consider independent groups. QTL with major effects were defined as the QTL with effects larger than the 75 % of the maximum, and QTL with minor effect were defined as the remaining QTL. The residual term was obtained by sampling from a normal distribu tion, N(0, σ^2^_e_), where σ^2^_e_ = (1- *h* ^*2*^)σ^2^_g_/ *h* ^*2*^ and σ^2^_g_ was the variance of the realized *g*. One vector for the combinations of number of QTL (i.e. 25 and 50), different heritabilities (i.e. 0.2, 0.4, 0.6, 0.7, 0.9), and for each one of 500 iterations was created. Then, we created missing data at random, imputed (i.e. G_*NImp,*_ G_*MVN-EM*_, G_*Mean*_ and G_*RF*_) and pursued the GWAS analysis with each combination of genotypic matrix, evaluating power and false positive rate (for the general approach see Fig. 1A.1).

For the ascertainment bias evaluation, we first created the missing data and then simulated the QTL on top of each matrix: not-imputed marker score (Y_sim-*NImp*_), imputed with MVN-EM [10] marker score (Y_sim-*MVN-EM*_), imputed by the mean marker score (Y_sim-*Mean*_) and imputed with RF [10] marker score (Y_sim-*RF*_). Finally, we performed the GWAS analysis with each genotypic marker score (i.e. G_*NImp,*_ G_*MVN-EM*_, G_*Mean*_ and G_*RF*_) and for each phenotypic vector (i.e. Y_sim-*NImp*_, Y_sim-*MVN-EM*_, Y_sim-*Mean*_ and Y_sim-*RF*_, for the general approach see Fig. 1A.2). We then compared the power and false positive rate.

For evaluating GWAS performance based on simulated phenotypes with the wheat GBS panel (Fig 1b) data we first created vectors of phenotypic values (i.e. Y_sim-*NImp*_, Y_sim-*MVN-EM*_, Y_sim-*Mean*_ and Y_sim-*RF*_). Each phenotypic vector was simulated for different number of QTL (i.e. 25 and 50), different heritabilities (i.e. 0.2, 0.4, 0.6, 0.7, 0.9) as in the previous section. In order to avoid collinearity, LD blocks were defined as the bins in each chromosome and a marker chosen at random within each LD block was considered a QTL. One vector for each combination of the parameters and for each one of 500 iterations was created. We performed the simulations in R statistical software [41].

### GWAS analysis

For the GWAS analysis, the mixed model described by [43] was used: y = Xβ + Qv + Zu + e, where y is the phenotypic vector (n x 1) with *n* the total number of lines, X is a (n x m) SNPs matrix with *m* the number of SNPs coded as described before {NA, 1, -1}, β is a (m x 1) vector of allelic effects to be estimated, Q is a (n x q) incidence matrix with *q* origin’s groups, v is a (n x 1) populations fixed effect vector, Z is the genotypic incidence matrix, u is the vector of random background polygenic effects, u ~ N(0, Aσ^2^_g_), where *A* is the realized additive relationship matrix obtained with the *A.mat* function from package *rrBLUP* [36] in R statistical software [35] and e is the residual error, e ~ N(0, σ^2^_e_). For each *Y*_*sim*_ *,* we used the 4 genotypic marker score to recover the QTL (i.e. G_*NImp,*_ G_*MVN-EM*_, G_*Mean*_ and G_*RF*_). We performed the analysis for three different thresholds (*threshold*) to define markers as significant: (1) Bonferroni correction, (2) Bonferroni correction using the effective number of markers, Li&Ji method [38], and (3) a liberal threshold of α = 0.01. GWAS analysis was accomplished with *GWAS* function from *rrBLUP* package [42] in R statistical software [41–45]. We defined as true positives (TP) the number of bins with a QTL and at least one significant marker; false positives (FP) the number of bins with no QTL and at least one significant marker; true negatives (TN) the number of bins with no QTL and no significant markers, and false negatives (FN) the number of bins with QTL and no significant markers. We evaluated power (*PO* = TP/(TP + FN)) and false positive rate (*FPR* = FP/ (FP + TN)) [39] for QTL detection. We evaluated performance for QTL of major and minor effect.

## Additional files

Additional file 1: Figure S1. Power (*PO*) and false positives rate (*FPR*) for major and minor QTL with 25 QTL, for the golden standard form barley, with a Bonferroni threshold. Each parameter was calculated for the combinations of: heritabilties (*h* ^*2*^), a marker score matrix to simulate the QTL (i.e. Y_sim-*NoNA*_), and marker score matrices to perform the GWAS analysis (i.e. G_*NImp,*_ G_*MVN-EM*_ and G_*Mean*_). (PDF 28 KB)
Additional file 2: Figure S2. Power (*PO*) and false positives rate (*FPR*) for major and minor QTL with 25 QTL, for the golden standard from barley, with α = 0.01 threshold. Each parameter was calculated for the combinations of: number of QTL (*q*), heritabilties (*h* ^*2*^), a marker score matrix to simulate the QTL (i.e. Y_sim-*NoNA*_), and marker score matrices to perform the GWAS analysis (i.e. G_*NImp,*_ G_*MVN-EM*_ and G_*Mean*_). (PDF 28 KB)
Additional file 3: Figure S3. Power (*PO*) and false positives rate (*FPR*) with 25 QTL, for major and minor QTL for ascertainment bias in imputation performance comparison in barley, with a Bonferroni threshold. Each parameter was calculated for the combinations of: heritabilties (*h* ^*2*^), marker score matrices to simulate the QTL (i.e. Y_sim-*NImp*_, Y_sim-*MVN-EM*_ and Y_sim-*Mean*_), and marker score matrices to perform the GWAS analysis (i.e. G_*NImp,*_ G_*MVN-EM*_ and G_*Mean*_). (PDF 34 KB)
Additional file 4: Figure S4. Power (*PO*) and false positives rate (*FPR*) with 25 QTL, for major and minor QTL for ascertainment bias in imputation performance comparison in barley, with a α = 0.01 threshold. Each parameter was calculated for the combinations of: heritabilties (*h* ^*2*^), marker score matrices to simulate the QTL (i.e. Y_sim-*NImp*_, Y_sim-*MVN-EM*_ and Y_sim-*Mean*_), and marker score matrices to perform the GWAS analysis (i.e. G_*NImp,*_ G_*MVN-EM*_ and G_*Mean*_). (PDF 34 KB)
Additional file 5: Figure S5. Power (*PO*) and false positives rate (*FPR*) with 25 QTL and 25 % missing rate, for major and minor QTL to evaluate the GWAS performance based on simulated matrix with a Bonferroni threshold corrected by the effective number of independent markers. Each parameter was calculated for the combinations of: heritabilties (*h* ^*2*^), marker score matrices to simulate the QTL (i.e. Y_sim-*NImp*_, Y_sim-*MVN-EM,*_ Y_sim-*Mean*_ and Y_sim-*RF*_), and marker score matrices to perform the GWAS analysis (i.e. G_*NImp,*_ G_*MVN-EM,*_ G_*Mean*_ and G_*RF*_). (PDF 156 KB)
Additional file 6: Figure S6. Power (*PO*) and false positives rate (*FPR*) with 25 QTL and 35 % missing rate, for major and minor QTL to evaluate the GWAS performance based on simulated matrix with a Bonferroni threshold corrected by the effective number of independent markers. Each parameter was calculated for the combinations of: heritabilties (*h* ^*2*^), marker score matrices to simulate the QTL (i.e. Y_sim-*NImp*_, Y_sim-*MVN-EM,*_ Y_sim-*Mean*_ and Y_sim-*RF*_), and marker score matrices to perform the GWAS analysis (i.e. G_*NImp,*_ G_*MVN-EM,*_ G_*Mean*_ and G_*RF*_). (PDF 156 KB)
Additional file 7: Figure S7. Power (*PO*) and false positives rate (*FPR*) with 25 QTL and 50 % missing rate, for major and minor QTL to evaluate the GWAS performance based on simulated matrix with a Bonferroni threshold. Each parameter was calculated for the combinations of: heritabilties (*h* ^*2*^), marker score matrices to simulate the QTL (i.e. Y_sim-*NImp*_, Y_sim-*MVN-EM*_ and Y_sim-*Mean*_), and marker score matrices to perform the GWAS analysis (i.e. G_*NImp,*_ G_*MVN-EM*_ and G_*Mean*_). (PDF 35 KB)
Additional file 8: Figure S8. Power (*PO*) and false positives rate (*FPR*) with 25 QTL and 50 % missing rate, for major and minor QTL to evaluate the GWAS performance based on simulated matrix with a α = 0.01 threshold. Each parameter was calculated for the combinations of: heritabilties (*h* ^*2*^), marker score matrices to simulate the QTL (i.e. Y_sim-*NImp*_, Y_sim-*MVN-EM*_ and Y_sim-*Mean*_), and marker score matrices to perform the GWAS analysis (i.e. G_*NImp,*_ G_*MVN-EM*_ and G_*Mean*_). (PDF 36 KB)
Additional file 9: Figure S9. QQ plots of the p-values from the GWAS analysis from real phenotype wheat data with 25 % missing rate and a Bonferroni threshold corrected by the effective number of independent markers. For each trait measured and each marker score matrix evaluated, a qq-plot of the *p*-values resulted form the GWAS analysis is presented. The marker score matrices were: *NImp* (not imputed) in turquoise*, Mean* (mean imputed) in green, *MVN-EM* (Multivariate Normal Expectation Maximization method) in coral and *RF* (Random Forest method) in orchid. The phenotype traits are: DH, days to heading; PH, Plant Height; SPM, Spikes Per Square Meter; TKW, Thousands Kernel Weight. (PDF 359 KB)
Additional file 10: Figure S10. QQ plots of the p-values from the GWAS analysis from real phenotype wheat data with 35 % missing rate and a Bonferroni threshold corrected by the effective number of independent markers. For each trait measured and each marker score matrix evaluated, a qq-plot of the p-values resulted form the GWAS analysis is presented. The marker score matrices were: *NImp* (not imputed) in turquoise*, Mean* (mean imputed) in green, *MVN-EM* (Multivariate Normal Expectation Maximization method) in coral and *RF* (Random Forest method) in orchid. The phenotype traits are: DH, days to heading; PH, Plant Height; SPM, Spikes Per Square Meter; TKW, Thousands Kernel Weight. (PDF 418 KB)
Additional file 11: Figure S11. Manhattan plots of the GWAS analysis for real phenotype wheat data with 25 % missing rate and a Bonferroni threshold corrected by the effective number of independent markers. For each trait measured and each marker score matrix evaluated, a manhattan plot of the GWAS analysis is presented. The phenotype traits are: DH, Days to Heading; PH, Plant Height; SPM, Spikes Per Square Meter; TKW, Thousands Kernel Weight. The marker score matrices were: *NImp* (not imputed)*, Mean* (mean imputed), *MVN-EM* (Multivariate Normal Expectation Maximization method) and *RF* (Random Forest method). QTL detected by the *NImp* matrix are in turquoise, QTL detected exclusively by the *MVN-EM* matrix are in coral, QTL detected exclusively by the *Mean* matrix are in green, and QTL detected exclusively by the *RF* matrix are in orchid. (PDF 539 KB)
Additional file 12: Figure S12. Manhattan plots of the GWAS analysis for real phenotype wheat data with 35 % missing rate and a Bonferroni threshold corrected by the effective number of independent markers. For each trait measured and each marker score matrix evaluated, a manhattan plot of the GWAS analysis is presented. The phenotype traits are: DH, Days to Heading; PH, Plant Height; SPM, Spikes Per Square Meter; TKW, Thousands Kernel Weight. The marker score matrices were: *NImp* (not imputed)*, Mean* (mean imputed), *MVN-EM* (Multivariate Normal Expectation Maximization method) and *RF* (Random Forest method). QTL detected by the *NImp* matrix are in turquoise, QTL detected exclusively by the *MVN-EM* matrix are in coral, QTL detected exclusively by the *Mean* matrix are in green, and QTL detected exclusively by the *RF* matrix are in orchid. (PDF 745 KB)
Additional file 13: Figure S13. Boxplots of false positives rate (*FPR*) for major and minor QTL with 25 QTL, for the golden standard form barley, with a Bonferroni threshold corrected by the effective number of independent markers. Each parameter was calculated for the combinations of: heritabilties (*h* ^*2*^), a marker score matrix to simulate the QTL (i.e. Y_sim-*NoNA*_), and marker score matrices to perform the GWAS analysis (i.e. G_*NImp,*_ G_*MVN-EM,*_ G_*Mean*_ and G_*RF*_). (PDF 110 KB)
Additional file 14: Figure S14. Boxplots of false positives rate (*FPR*) with 25 QTL, for major and minor QTL for ascertainment bias in imputation performance comparison in barley, with a Bonferroni threshold corrected by the effective number of independent markers. Each parameter was calculated for the combinations of: heritabilties (*h* ^*2*^), marker score matrices to simulate the QTL (i.e. Y_sim-*NImp*_, Y_sim-*MVN-EM,*_ Y_sim-*Mean*_ and Y_sim-*RF*_), and marker score matrices to perform the GWAS analysis (i.e. G_*NImp,*_ G_*MVN-EM,*_ G_*Mean*_ and G_*RF*_). (PDF 139 KB)
Additional file 15: Figure S15. Boxplots of false positives rate (*FPR*) with 25 QTL and 50 % missing rate, for major and minor QTL to evaluate the GWAS performance based on simulated matrix with a Bonferroni threshold corrected by the effective number of independent markers. Each parameter was calculated for the combinations of: heritabilties (*h* ^*2*^), marker score matrices to simulate the QTL (i.e. Y_sim-*NImp*_, Y_sim-*MVN-EM,*_ Y_sim-*Mean*_ and Y_sim-*RF*_), and marker score matrices to perform the GWAS analysis (i.e. G_*NImp,*_ G_*MVN-EM,*_ G_*Mean*_ and G_*RF*_). (PDF 144 KB )
Additional file 16: Figure S16. Boxplots of false positives rate (*FPR*) with 25 QTL and 25 % missing rate, for major and minor QTL to evaluate the GWAS performance based on simulated matrix with a Bonferroni threshold corrected by the effective number of independent markers. Each parameter was calculated for the combinations of: heritabilties (*h* ^*2*^), marker score matrices to simulate the QTL (i.e. Y_sim-*NImp*_, Y_sim-*MVN-EM,*_ Y_sim-*Mean*_ and Y_sim-*RF*_), and marker score matrices to perform the GWAS analysis (i.e. G_*NImp,*_ G_*MVN-EM,*_ G_*Mean*_ and G_*RF*_). (PDF 132 KB)
Additional file 17: Figure S17. Boxplots of false positives rate (*FPR*) with 25 QTL and 35 % missing rate, for major and minor QTL to evaluate the GWAS performance based on simulated matrix with a Bonferroni threshold corrected by the effective number of independent markers. Each parameter was calculated for the combinations of: heritabilties (*h* ^*2*^), marker score matrices to simulate the QTL (i.e. Y_sim-*NImp*_, Y_sim-*MVN-EM,*_ Y_sim-*Mean*_ and Y_sim-*RF*_), and marker score matrices to perform the GWAS analysis (i.e. G_*NImp,*_ G_*MVN-EM,*_ G_*Mean*_ and G_*RF*_). (PDF 143 KB)
Additional file 18: Barley SNP marker score array. Genotypes are presented as rows and SNPs as columns. (txt 278 kb)
Additional file 19: Barley SNP marker map. Markers, chromosome and position (cM) are presented as columns. (txt 18 kb)
Additional file 20: Wheat GBS dataset with 50 % missing information. Genotypes are presented as rows and SNPs as columns. (txt 16.3 Mb)
Additional file 21: Wheat GBS bin map for 50 % coverage. Markers, chromosome and position (bins) are presented as columns. (txt 287 kb)
Additional file 22: Wheat GBS dataset with 25 % missing information. Genotypes are presented as rows and SNPs as columns. (txt 16.3 Mb)
Additional file 23: Wheat GBS bin map for 25 % coverage. Markers, chromosome and position (bins) are presented as columns. (txt 287 kb)
Additional file 24: Wheat GBS dataset with 35 % missing information. Genotypes are presented as rows and SNPs as columns. (txt 16.3 Mb)
Additional file 25: Wheat GBS bin map for 35 % coverage. Markers, chromosome and position (bins) are presented as columns. (txt 287 kb)
Additional file 26: Best linear unbiased predictors (BLUPs) for each genotype for plant height. (txt 9 kb)
Additional file 27: Best linear unbiased predictors (BLUPs) for each genotype for days to heading. (txt 8 kb)
Additional file 28: Best linear unbiased predictors (BLUPs) for each genotype for thousands kernel weight. (txt 9 kb)
Additional file 29: Best linear unbiased predictors (BLUPs) for each genotype for spikes per square meter. (txt 9 kb)

## Abbreviations

BLUPs: Best linear unbiased predictors
BOPA: Barley oligonucleotide pool assay-1
CIMMYT: International maize and wheat improvement center
DH: Days to heading
FN: False negatives
FP: False positives
FPR: False positive rate
GBS: Genotype-by-sequencing
GS: Genome-wide selection
GWAS: Genome-wide analysis
ICARDA: International center for agricultural research in the dry areas
INIA: National institute of agricultural research
LD: Linkage disequilibrium
MAF: Minor allele frequency
MVN-EM: Multivariate normal expectation maximization
NGS: Next-generation sequencing
PH: Plant height
PO: Power
QTL: Quantitative trait loci
RF: Random forest
SNPs: Single-nucleotide polymorphism
SPM: Spikes per square meter
SR2011: Santa rosa 2011
TKW: Thousands kernel weight
TN: True negatives
TP: True positives
USDA: United States department of agriculture
