# Supplementary figures and images for: Ascertainment bias from imputation methods evaluation in wheat
Source: BMC Genomics. 2016 Oct 4;17:773. doi: 10.1186/s12864-016-3120-5 (PMC5050639; doi:10.1186/s12864-016-3120-5)

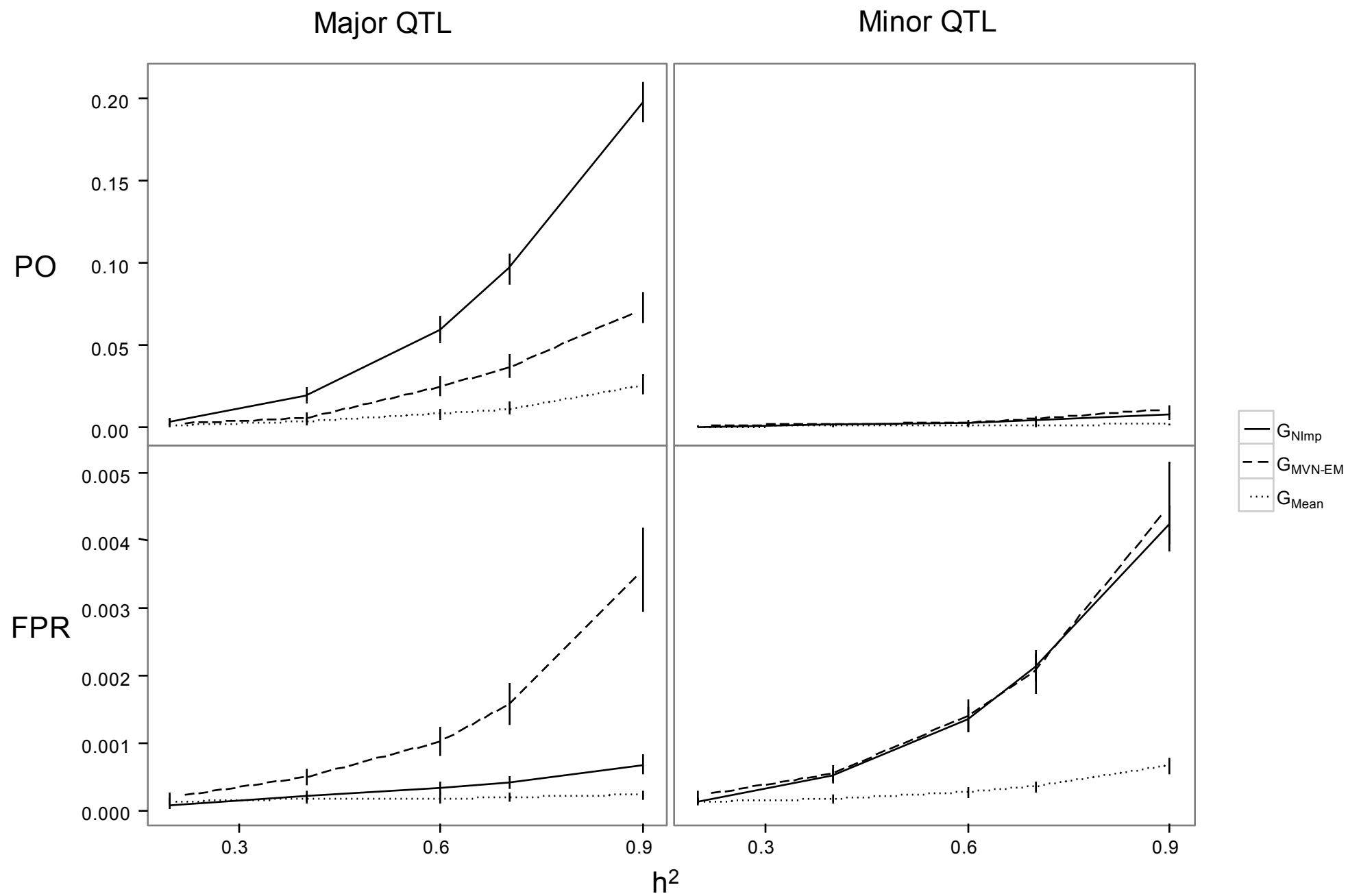

Supplement: Additional file 1: Figure S1. — Power (PO) and false positives rate (FPR) for major and minor QTL with 25 QTL, for the golden standard form barley, with a Bonferroni threshold. Each parameter was calculated for the combinations of: heritabilties (h 2), a marker score matrix to simulate the QTL (i.e. Ysim-NoNA), and marker score matrices to perform the GWAS analysis (i.e. GNImp, GMVN-EM and GMean). (PDF 28 KB) [file 12864_2016_3120_MOESM1_ESM.pdf]

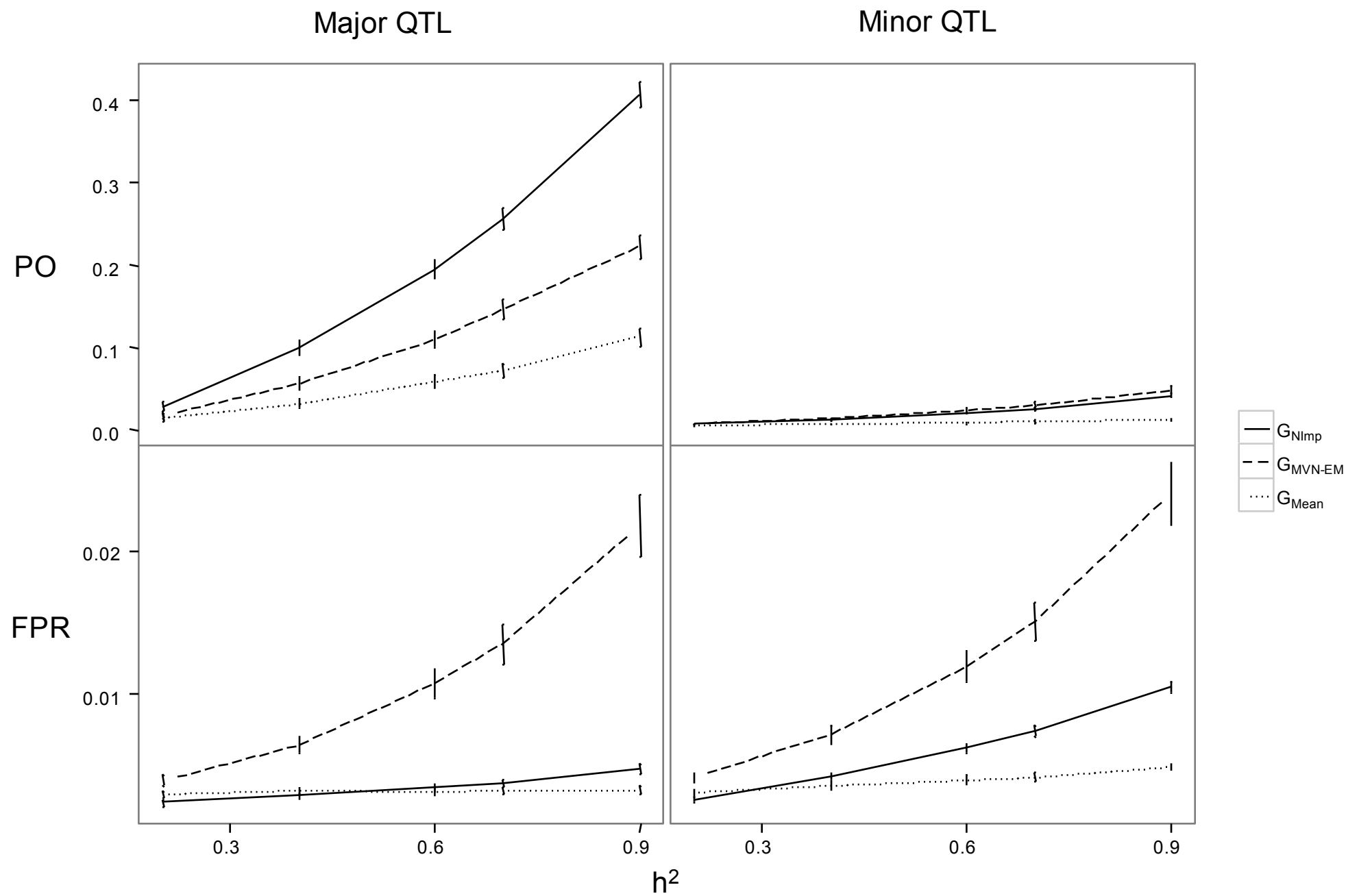

Supplement: Additional file 2: Figure S2. — Power (PO) and false positives rate (FPR) for major and minor QTL with 25 QTL, for the golden standard from barley, with α = 0.01 threshold. Each parameter was calculated for the combinations of: number of QTL (q), heritabilties (h 2), a marker score matrix to simulate the QTL (i.e. Ysim-NoNA), and marker score matrices to perform the GWAS analysis (i.e. GNImp, GMVN-EM and GMean). (PDF 28 KB) [file 12864_2016_3120_MOESM2_ESM.pdf]

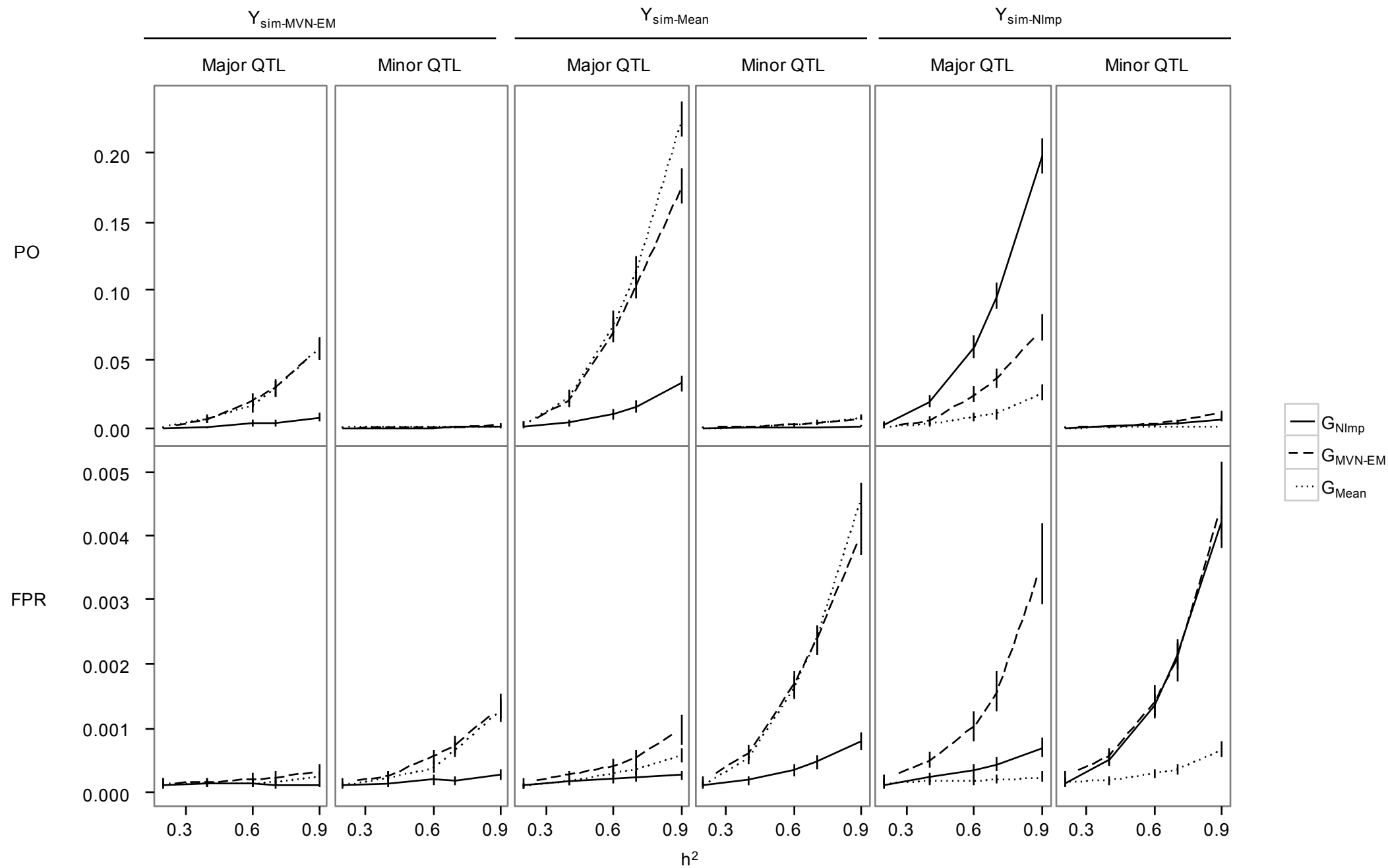

Supplement: Additional file 3: Figure S3. — Power (PO) and false positives rate (FPR) with 25 QTL, for major and minor QTL for ascertainment bias in imputation performance comparison in barley, with a Bonferroni threshold. Each parameter was calculated for the combinations of: heritabilties (h 2), marker score matrices to simulate the QTL (i.e. Ysim-NImp, Ysim-MVN-EM and Ysim-Mean), and marker score matrices to perform the GWAS analysis (i.e. GNImp, GMVN-EM and GMean). (PDF 34 KB) [file 12864_2016_3120_MOESM3_ESM.pdf]

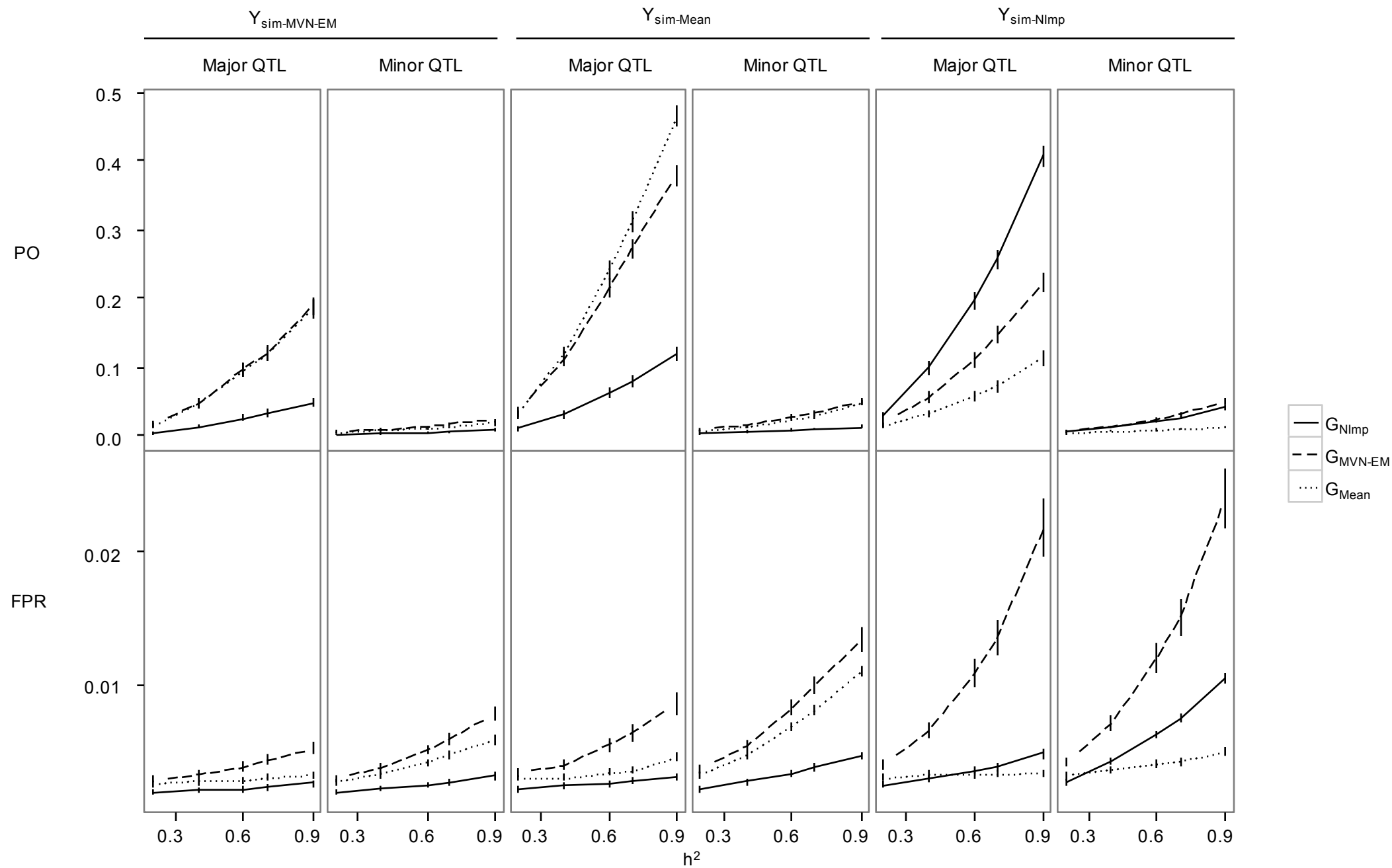

Supplement: Additional file 4: Figure S4. — Power (PO) and false positives rate (FPR) with 25 QTL, for major and minor QTL for ascertainment bias in imputation performance comparison in barley, with a α = 0.01 threshold. Each parameter was calculated for the combinations of: heritabilties (h 2), marker score matrices to simulate the QTL (i.e. Ysim-NImp, Ysim-MVN-EM and Ysim-Mean), and marker score matrices to perform the GWAS analysis (i.e. GNImp, GMVN-EM and GMean). (PDF 34 KB) [file 12864_2016_3120_MOESM4_ESM.pdf]

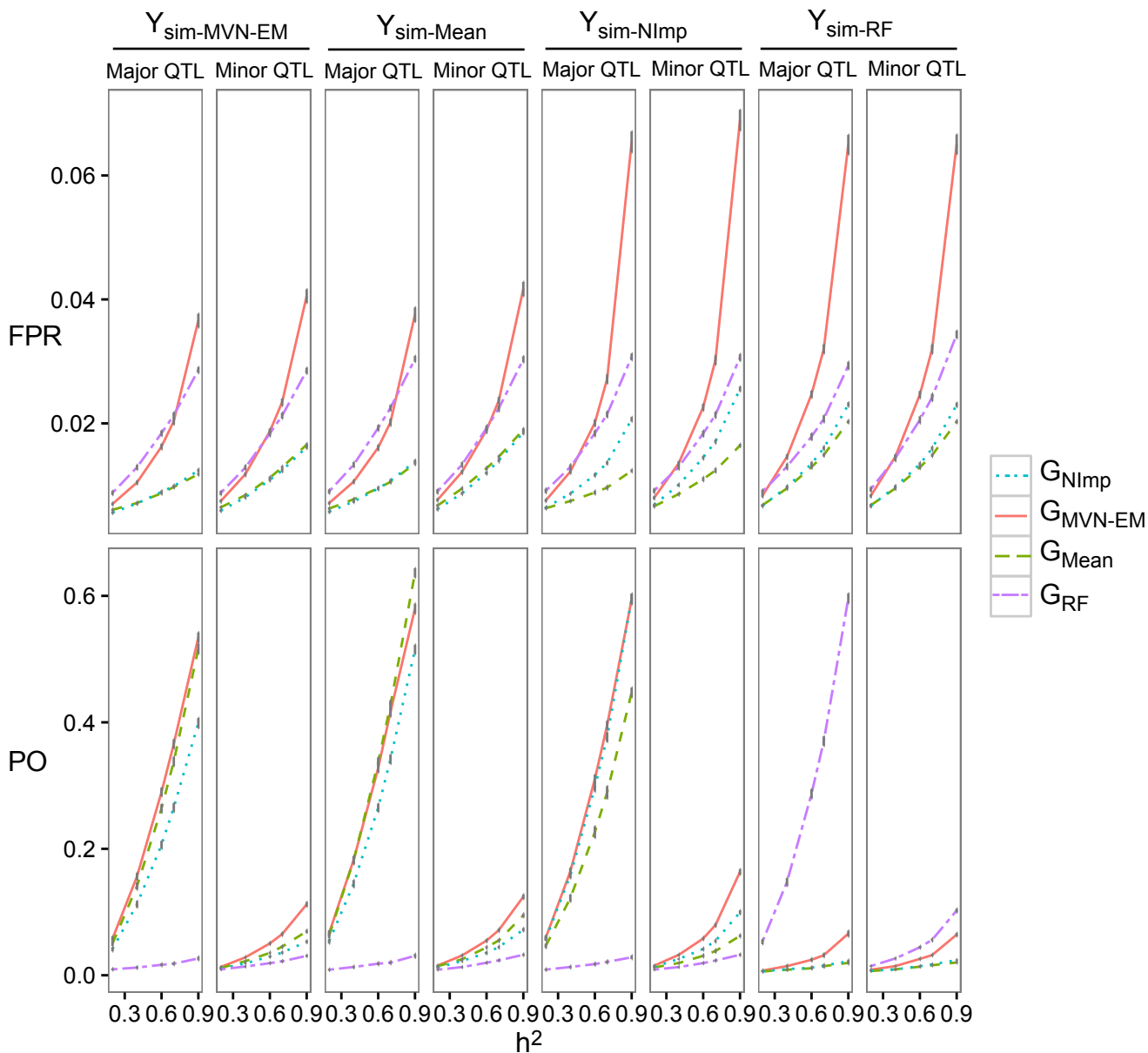

Supplement: Additional file 5: Figure S5. — Power (PO) and false positives rate (FPR) with 25 QTL and 25 % missing rate, for major and minor QTL to evaluate the GWAS performance based on simulated matrix with a Bonferroni threshold corrected by the effective number of independent markers. Each parameter was calculated for the combinations of: heritabilties (h 2), marker score matrices to simulate the QTL (i.e. Ysim-NImp, Ysim-MVN-EM, Ysim-Mean and Ysim-RF), and marker score matrices to perform the GWAS analysis (i.e. GNImp, GMVN-EM, GMean and GRF). (PDF 156 KB) [file 12864_2016_3120_MOESM5_ESM.pdf]

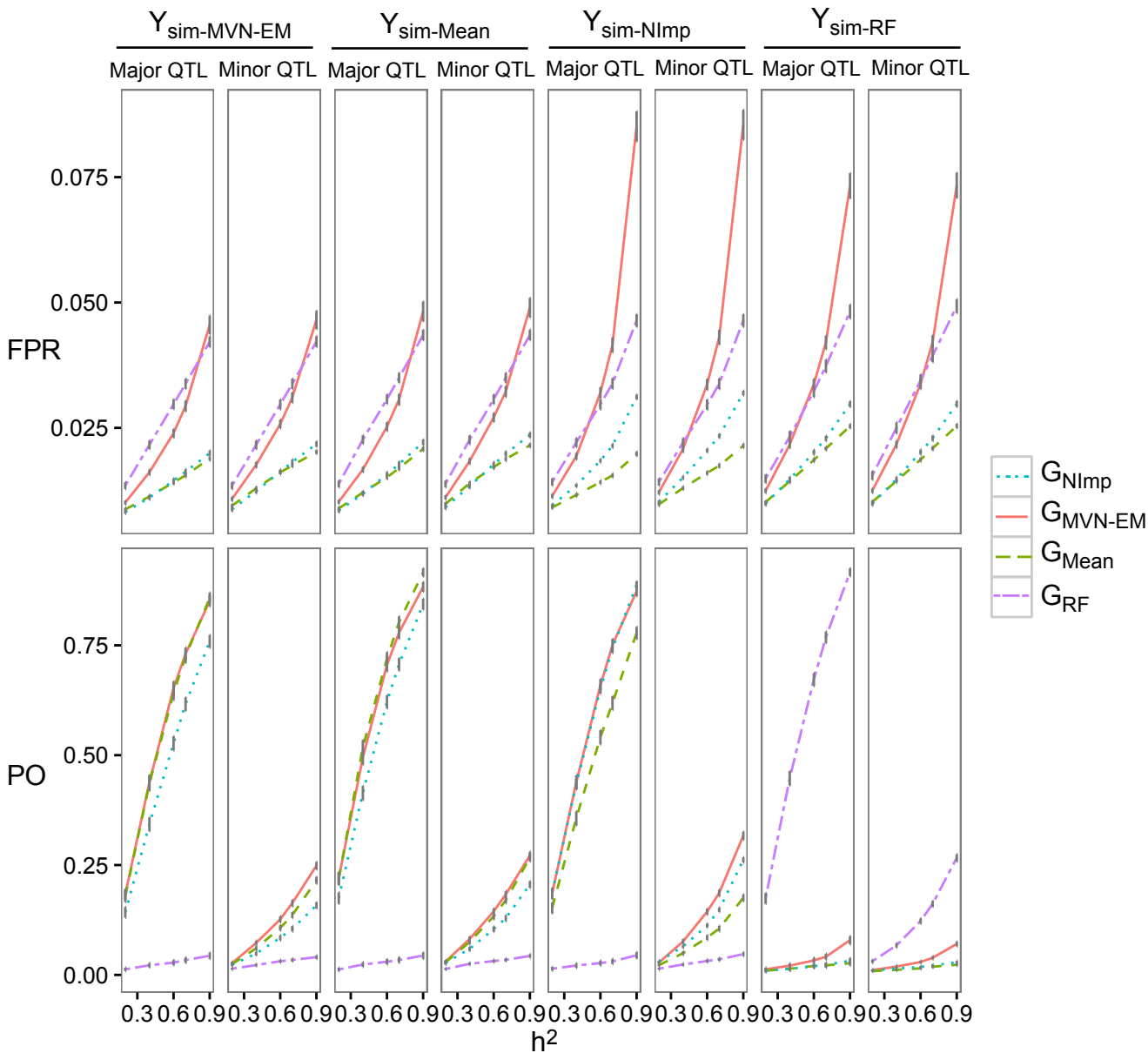

Supplement: Additional file 6: Figure S6. — Power (PO) and false positives rate (FPR) with 25 QTL and 35 % missing rate, for major and minor QTL to evaluate the GWAS performance based on simulated matrix with a Bonferroni threshold corrected by the effective number of independent markers. Each parameter was calculated for the combinations of: heritabilties (h 2), marker score matrices to simulate the QTL (i.e. Ysim-NImp, Ysim-MVN-EM, Ysim-Mean and Ysim-RF), and marker score matrices to perform the GWAS analysis (i.e. GNImp, GMVN-EM, GMean and GRF). (PDF 156 KB) [file 12864_2016_3120_MOESM6_ESM.pdf]

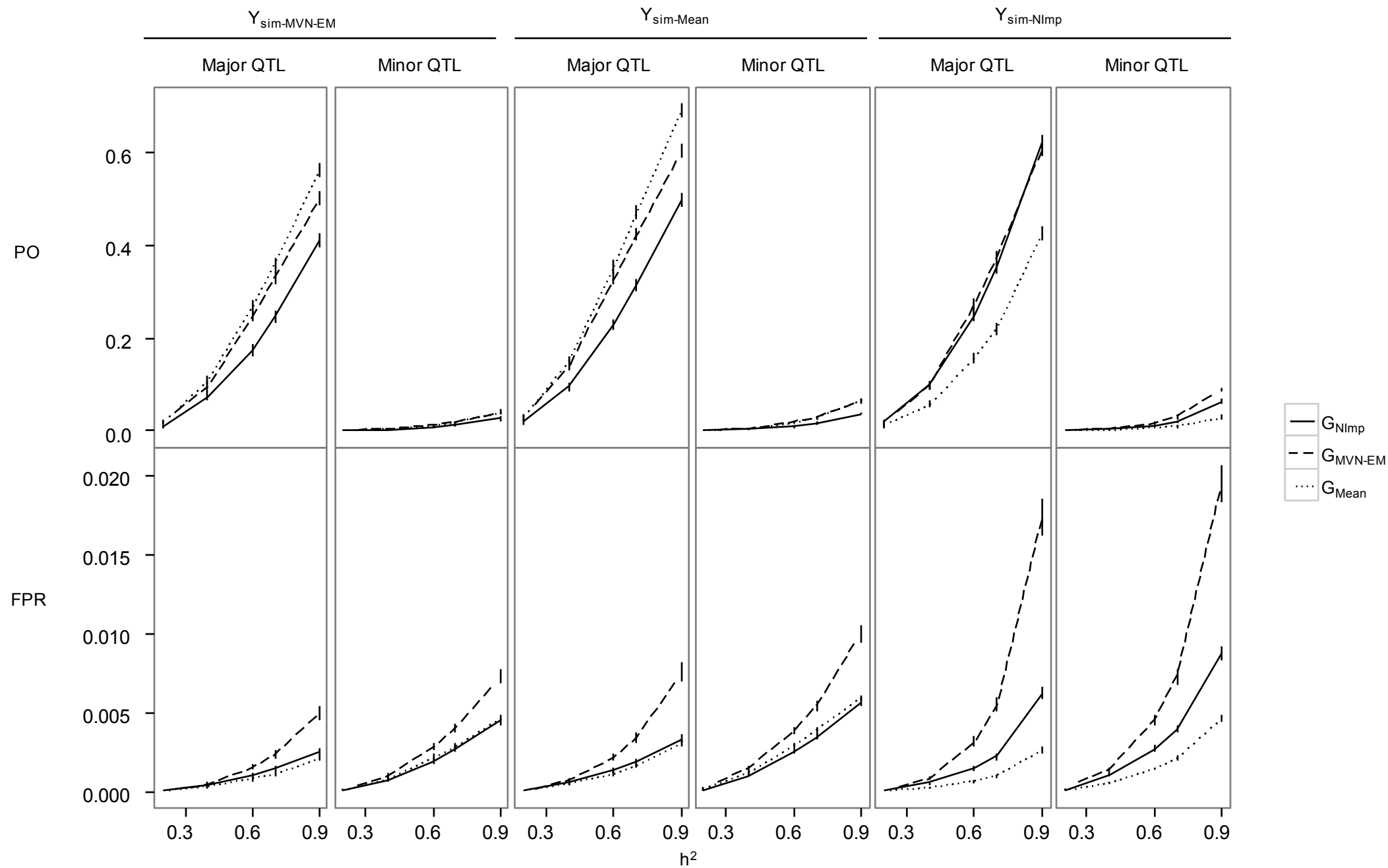

Supplement: Additional file 7: Figure S7. — Power (PO) and false positives rate (FPR) with 25 QTL and 50 % missing rate, for major and minor QTL to evaluate the GWAS performance based on simulated matrix with a Bonferroni threshold. Each parameter was calculated for the combinations of: heritabilties (h 2), marker score matrices to simulate the QTL (i.e. Ysim-NImp, Ysim-MVN-EM and Ysim-Mean), and marker score matrices to perform the GWAS analysis (i.e. GNImp, GMVN-EM and GMean). (PDF 35 KB) [file 12864_2016_3120_MOESM7_ESM.pdf]

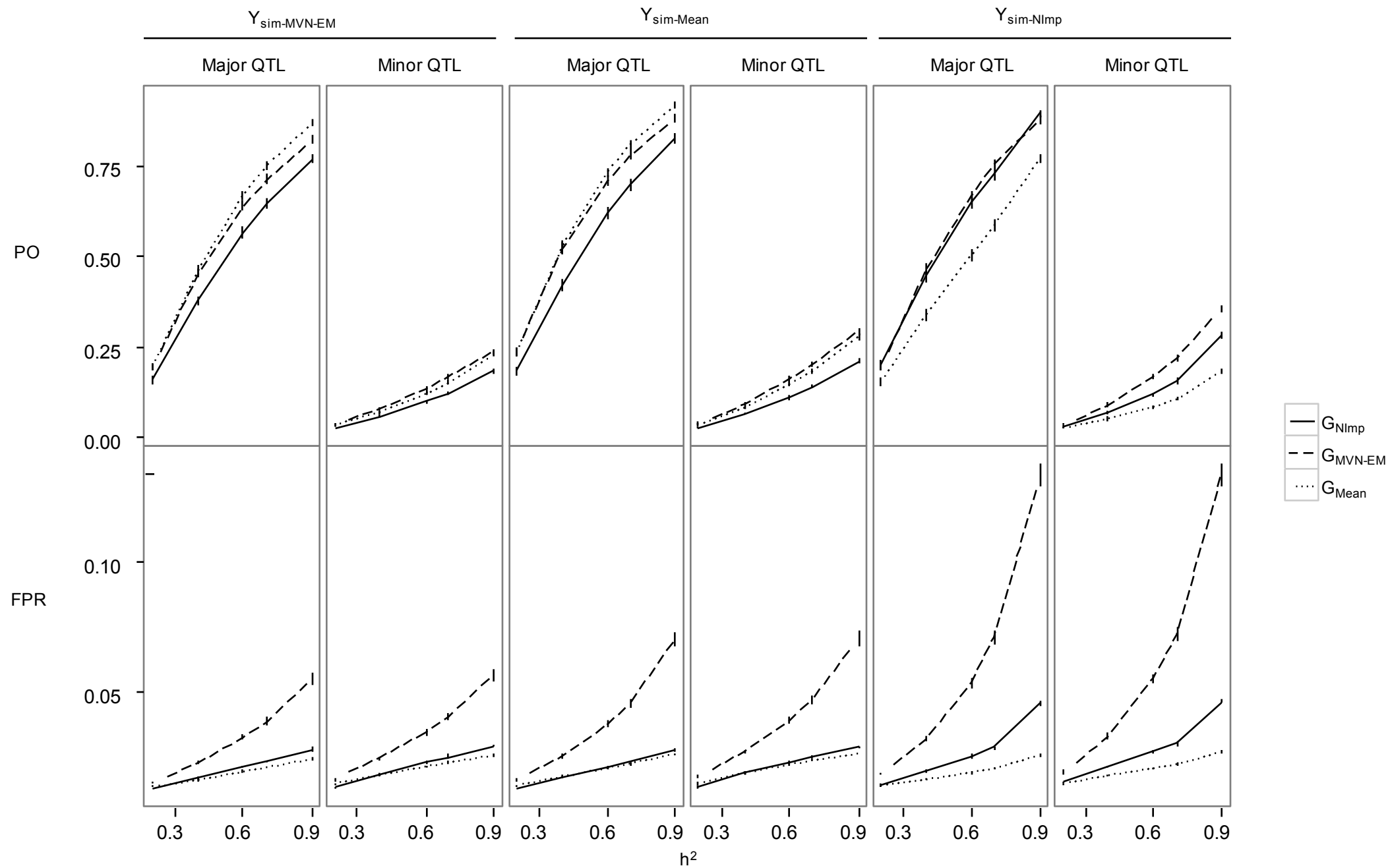

Supplement: Additional file 8: Figure S8. — Power (PO) and false positives rate (FPR) with 25 QTL and 50 % missing rate, for major and minor QTL to evaluate the GWAS performance based on simulated matrix with a α = 0.01 threshold. Each parameter was calculated for the combinations of: heritabilties (h 2), marker score matrices to simulate the QTL (i.e. Ysim-NImp, Ysim-MVN-EM and Ysim-Mean), and marker score matrices to perform the GWAS analysis (i.e. GNImp, GMVN-EM and GMean). (PDF 36 KB) [file 12864_2016_3120_MOESM8_ESM.pdf]

**TKW**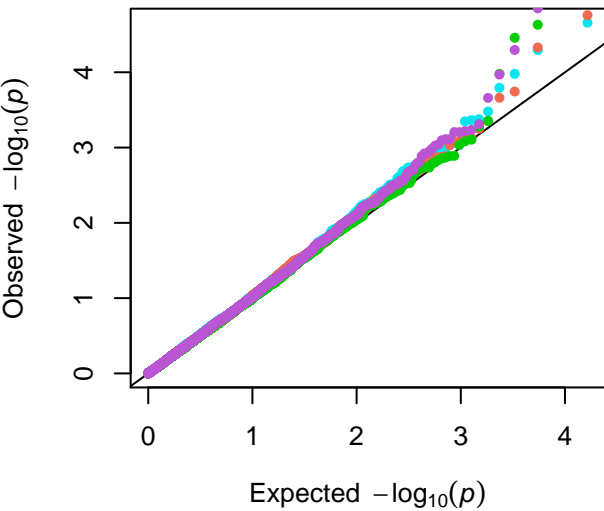**DH**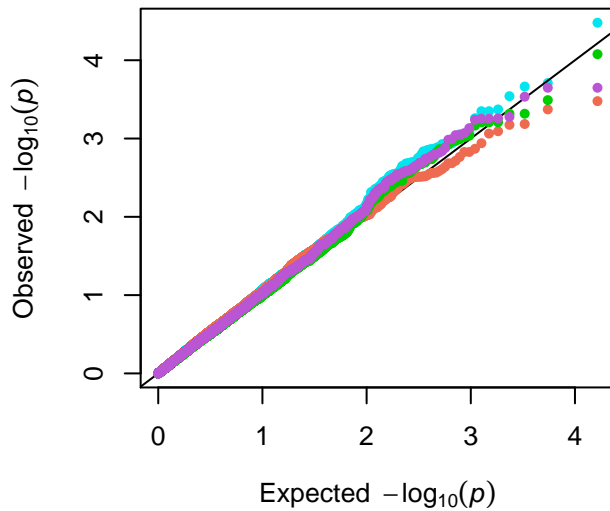**PH**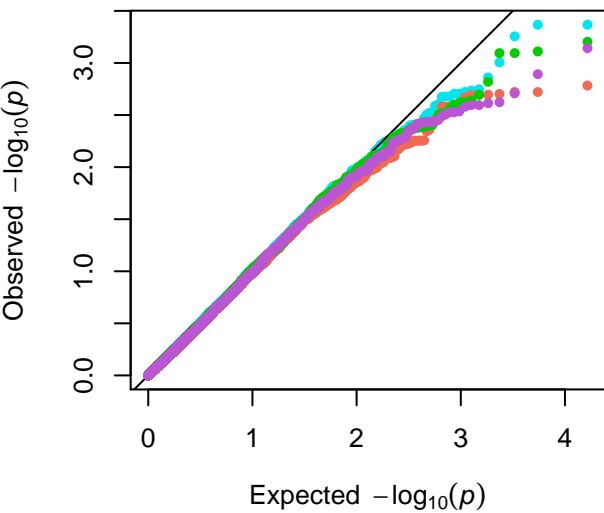**SPM**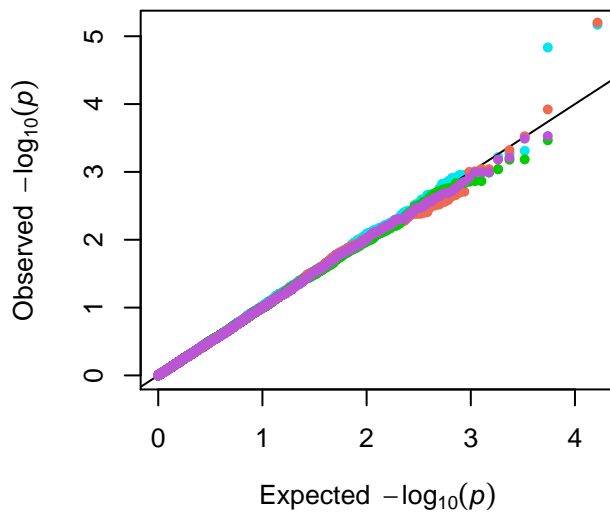

Supplement: Additional file 9: Figure S9. — QQ plots of the p-values from the GWAS analysis from real phenotype wheat data with 25 % missing rate and a Bonferroni threshold corrected by the effective number of independent markers. For each trait measured and each marker score matrix evaluated, a qq-plot of the p-values resulted form the GWAS analysis is presented. The marker score matrices were: NImp (not imputed) in turquoise, Mean (mean imputed) in green, MVN-EM (Multivariate Normal Expectation Maximization method) in coral and RF (Random Forest method) in orchid. The phenotype traits are: DH, days to heading; PH, Plant Height; SPM, Spikes Per Square Meter; TKW, Thousands Kernel Weight. (PDF 359 KB) [file 12864_2016_3120_MOESM9_ESM.pdf]

**TKW**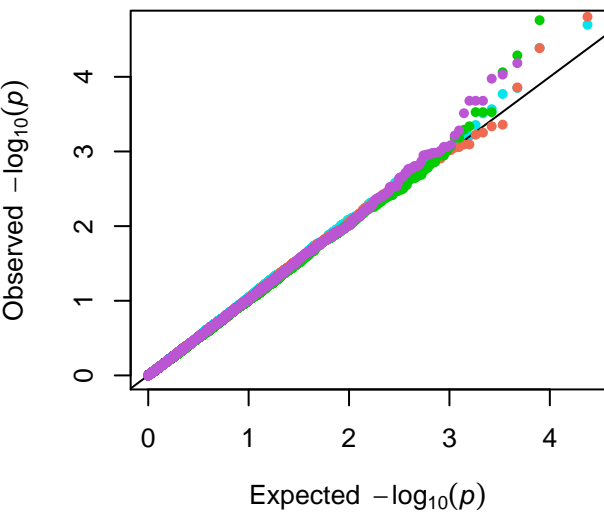**DH**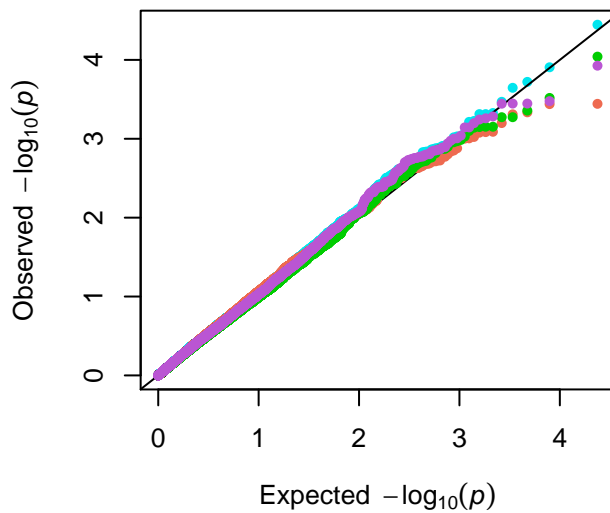**PH**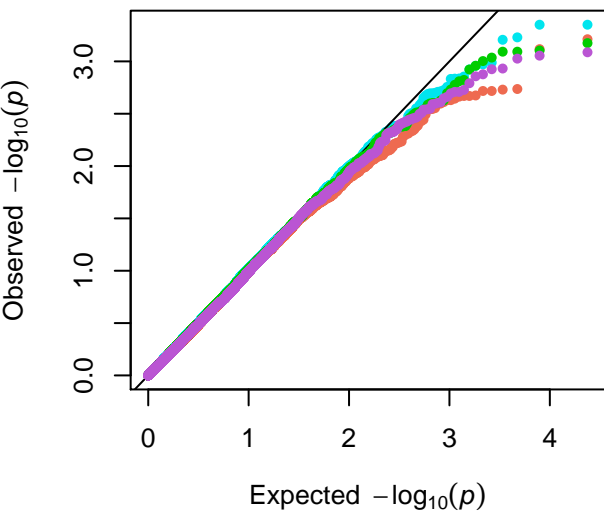**SPM**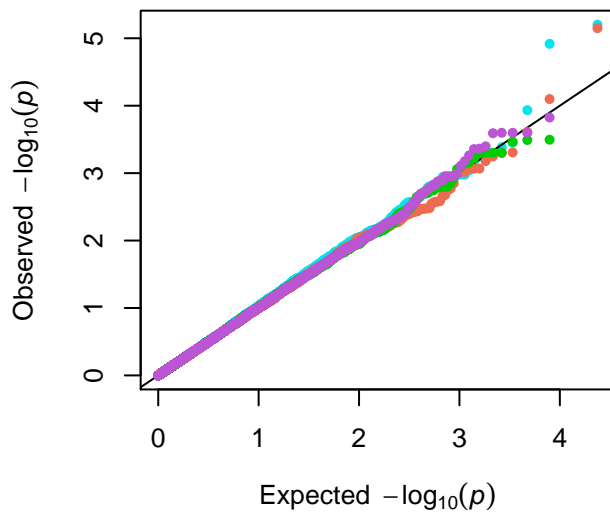

Supplement: Additional file 10: Figure S10. — QQ plots of the p-values from the GWAS analysis from real phenotype wheat data with 35 % missing rate and a Bonferroni threshold corrected by the effective number of independent markers. For each trait measured and each marker score matrix evaluated, a qq-plot of the p-values resulted form the GWAS analysis is presented. The marker score matrices were: NImp (not imputed) in turquoise, Mean (mean imputed) in green, MVN-EM (Multivariate Normal Expectation Maximization method) in coral and RF (Random Forest method) in orchid. The phenotype traits are: DH, days to heading; PH, Plant Height; SPM, Spikes Per Square Meter; TKW, Thousands Kernel Weight. (PDF 418 KB) [file 12864_2016_3120_MOESM10_ESM.pdf]

Major QTL

Minor QTL

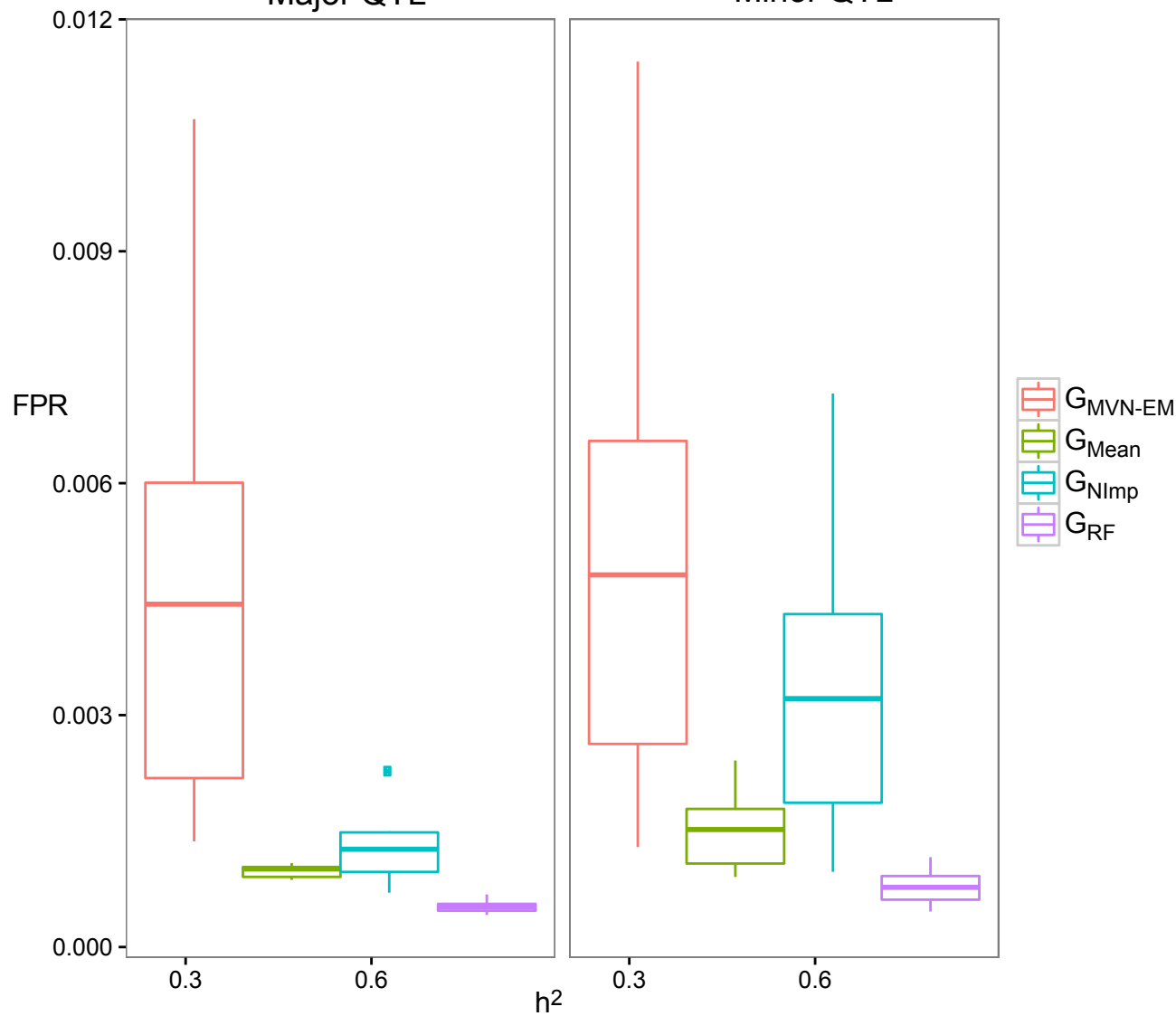

Supplement: Additional file 13: Figure S13. — Boxplots of false positives rate (FPR) for major and minor QTL with 25 QTL, for the golden standard form barley, with a Bonferroni threshold corrected by the effective number of independent markers. Each parameter was calculated for the combinations of: heritabilties (h 2), a marker score matrix to simulate the QTL (i.e. Ysim-NoNA), and marker score matrices to perform the GWAS analysis (i.e. GNImp, GMVN-EM, GMean and GRF). (PDF 110 KB) [file 12864_2016_3120_MOESM13_ESM.pdf]

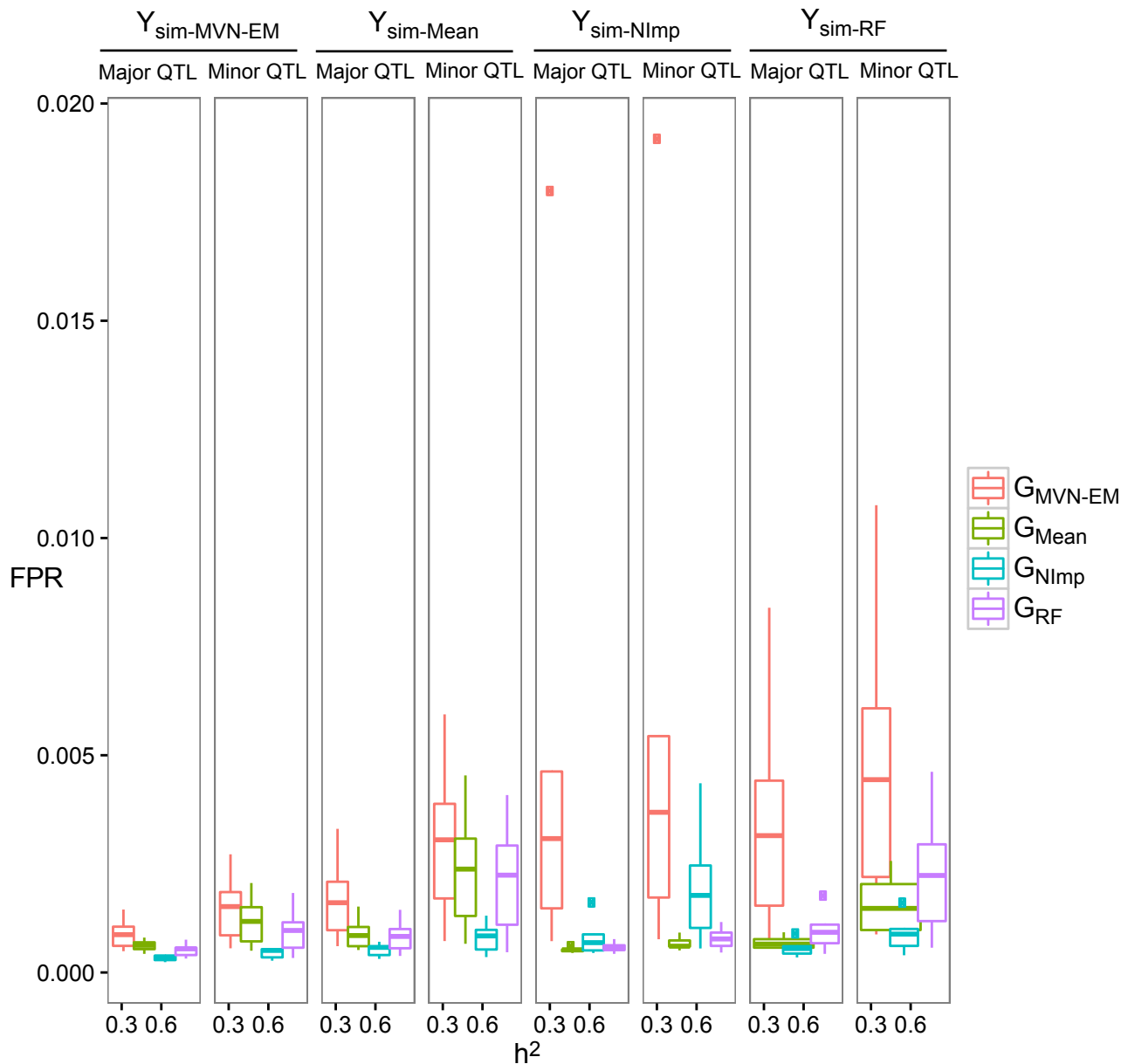

Supplement: Additional file 14: Figure S14. — Boxplots of false positives rate (FPR) with 25 QTL, for major and minor QTL for ascertainment bias in imputation performance comparison in barley, with a Bonferroni threshold corrected by the effective number of independent markers. Each parameter was calculated for the combinations of: heritabilties (h 2), marker score matrices to simulate the QTL (i.e. Ysim-NImp, Ysim-MVN-EM, Ysim-Mean and Ysim-RF), and marker score matrices to perform the GWAS analysis (i.e. GNImp, GMVN-EM, GMean and GRF). (PDF 139 KB) [file 12864_2016_3120_MOESM14_ESM.pdf]

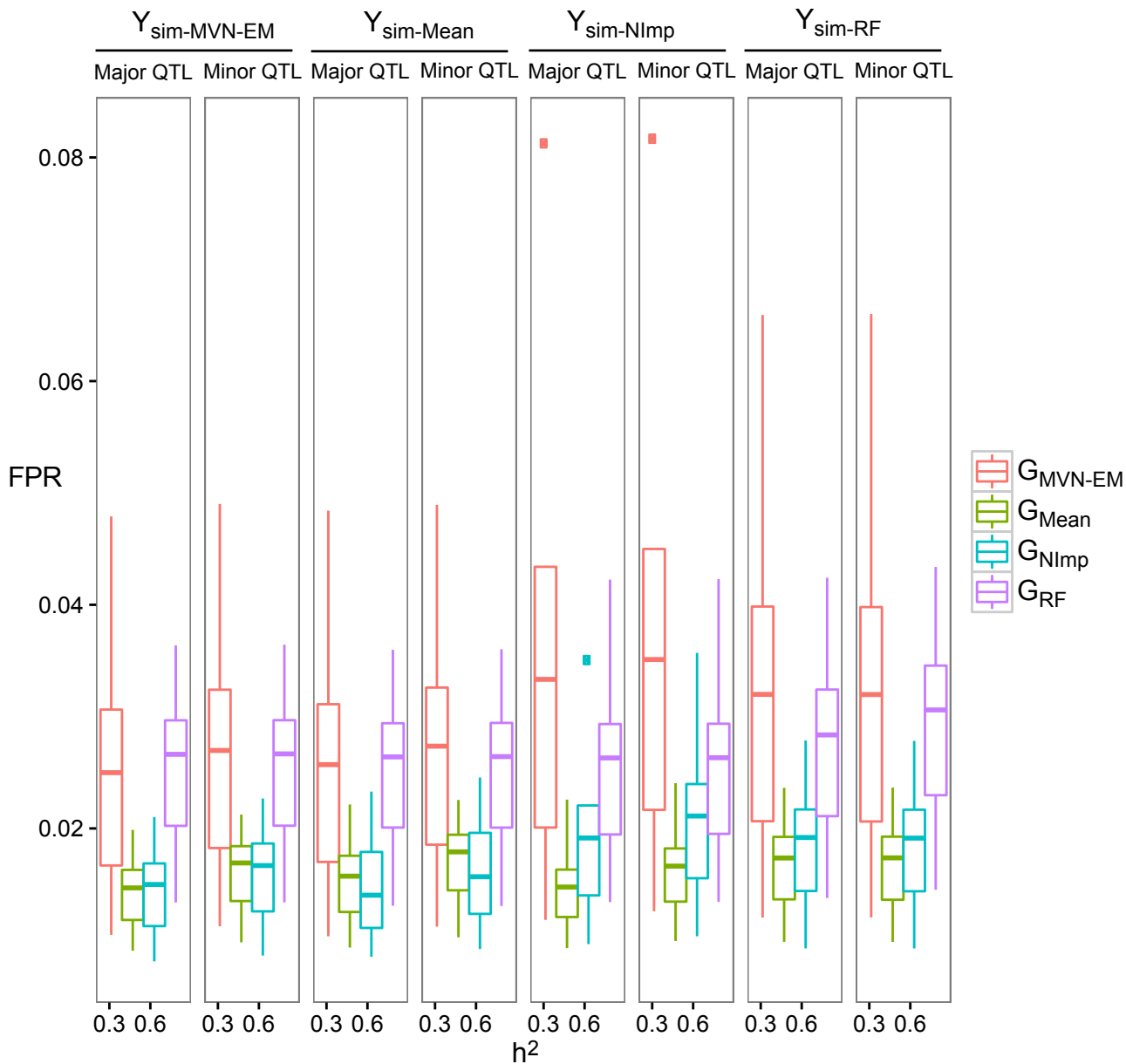

Supplement: Additional file 15: Figure S15. — Boxplots of false positives rate (FPR) with 25 QTL and 50 % missing rate, for major and minor QTL to evaluate the GWAS performance based on simulated matrix with a Bonferroni threshold corrected by the effective number of independent markers. Each parameter was calculated for the combinations of: heritabilties (h 2), marker score matrices to simulate the QTL (i.e. Ysim-NImp, Ysim-MVN-EM, Ysim-Mean and Ysim-RF), and marker score matrices to perform the GWAS analysis (i.e. GNImp, GMVN-EM, GMean and GRF). (PDF 144 KB ) [file 12864_2016_3120_MOESM15_ESM.pdf]

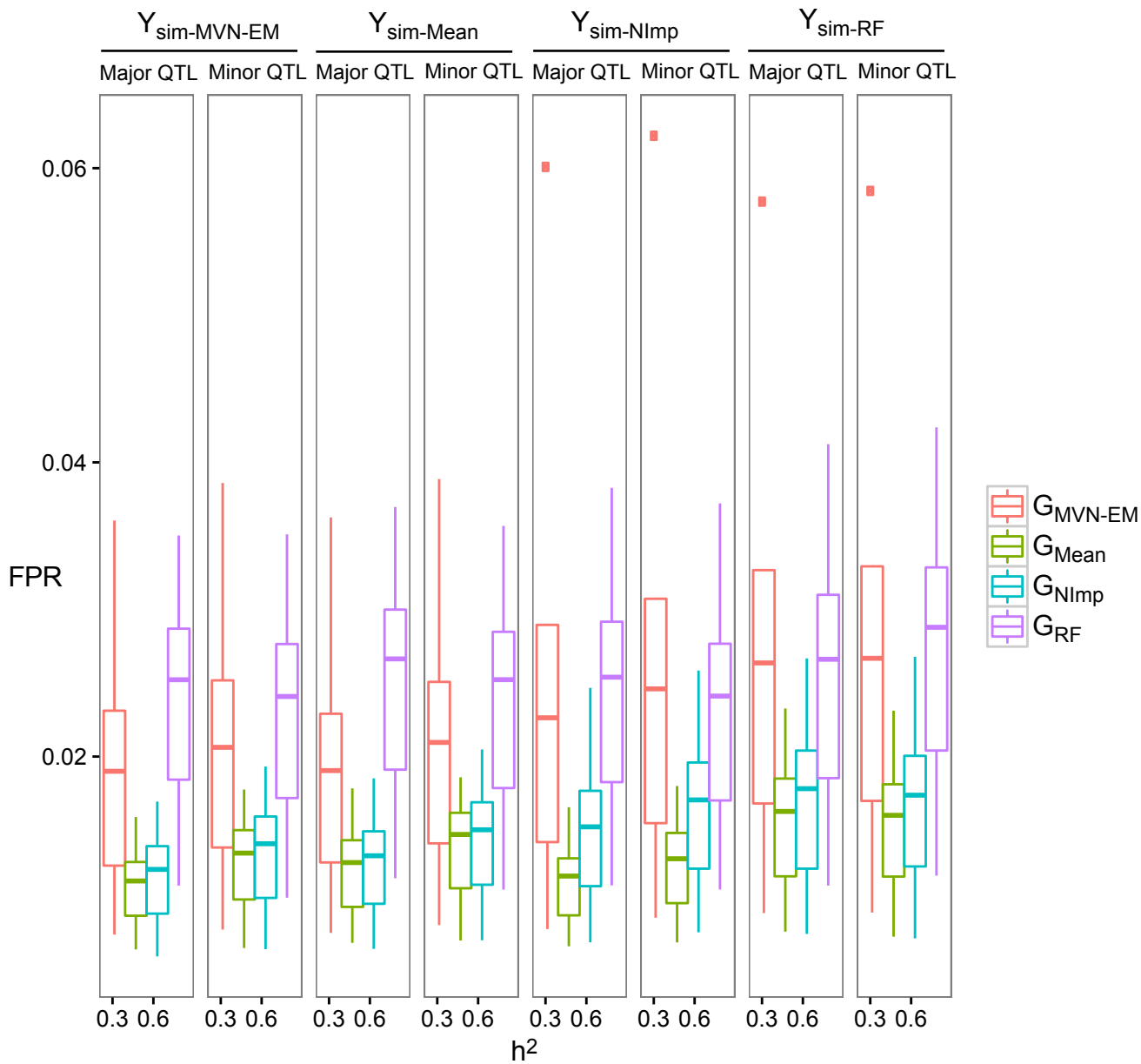

Supplement: Additional file 16: Figure S16. — Boxplots of false positives rate (FPR) with 25 QTL and 25 % missing rate, for major and minor QTL to evaluate the GWAS performance based on simulated matrix with a Bonferroni threshold corrected by the effective number of independent markers. Each parameter was calculated for the combinations of: heritabilties (h 2), marker score matrices to simulate the QTL (i.e. Ysim-NImp, Ysim-MVN-EM, Ysim-Mean and Ysim-RF), and marker score matrices to perform the GWAS analysis (i.e. GNImp, GMVN-EM, GMean and GRF). (PDF 132 KB) [file 12864_2016_3120_MOESM16_ESM.pdf]

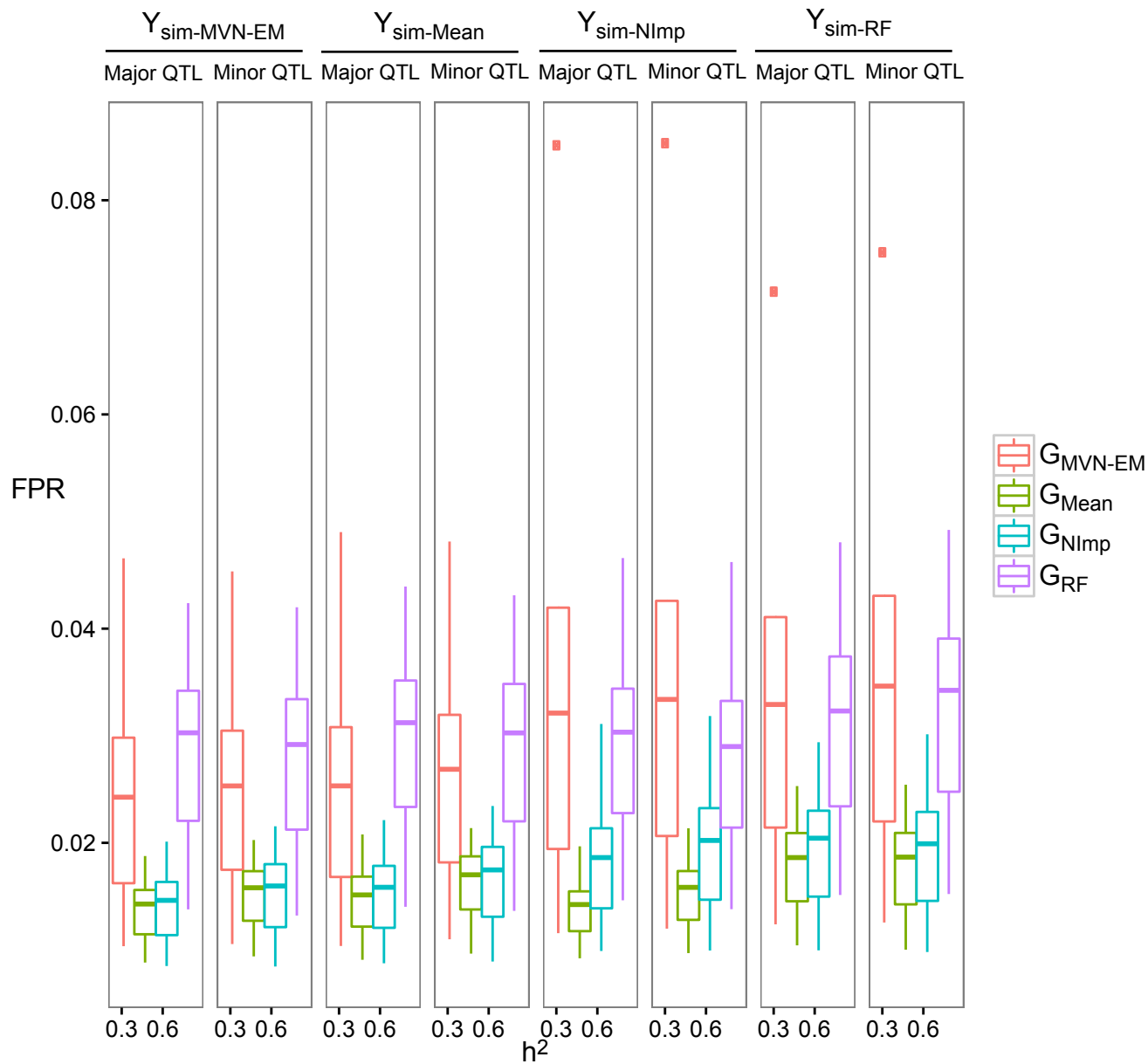

Supplement: Additional file 17: Figure S17. — Boxplots of false positives rate (FPR) with 25 QTL and 35 % missing rate, for major and minor QTL to evaluate the GWAS performance based on simulated matrix with a Bonferroni threshold corrected by the effective number of independent markers. Each parameter was calculated for the combinations of: heritabilties (h 2), marker score matrices to simulate the QTL (i.e. Ysim-NImp, Ysim-MVN-EM, Ysim-Mean and Ysim-RF), and marker score matrices to perform the GWAS analysis (i.e. GNImp, GMVN-EM, GMean and GRF). (PDF 143 KB) [file 12864_2016_3120_MOESM17_ESM.pdf]
