# Supplementary material for: Ascertainment bias from imputation methods evaluation in wheat
Source: BMC Genomics. 2016 Oct 4;17:773. doi: 10.1186/s12864-016-3120-5 (PMC5050639; doi:10.1186/s12864-016-3120-5)
Supplement: Additional file 12: Figure S12. — Manhattan plots of the GWAS analysis for real phenotype wheat data with 35 % missing rate and a Bonferroni threshold corrected by the effective number of independent markers. For each trait measured and each marker score matrix evaluated, a manhattan plot of the GWAS analysis is presented. The phenotype traits are: DH, Days to Heading; PH, Plant Height; SPM, Spikes Per Square Meter; TKW, Thousands Kernel Weight. The marker score matrices were: NImp (not imputed), Mean (mean imputed), MVN-EM (Multivariate Normal Expectation Maximization method) and RF (Random Forest method). QTL detected by the NImp matrix are in turquoise, QTL detected exclusively by the MVN-EM matrix are in coral, QTL detected exclusively by the Mean matrix are in green, and QTL detected exclusively by the RF matrix are in orchid. (PDF 745 KB) [file 12864_2016_3120_MOESM12_ESM.pdf]

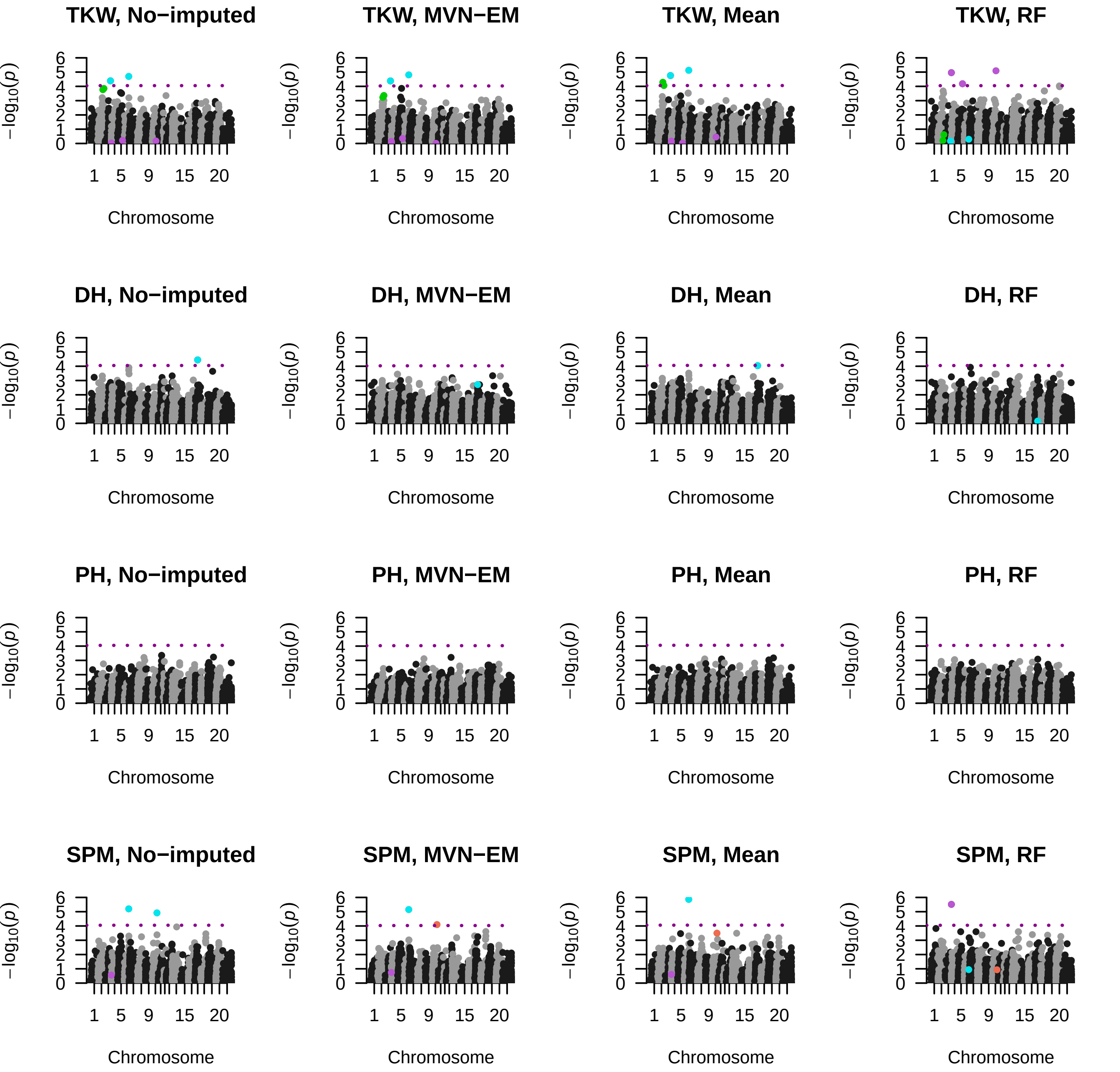
TKW, No−imputed
TKW, MVN−EM
TKW, Mean
TKW, RF
DH, No−imputed
DH, MVN−EM
DH, Mean
DH, RF
PH, No−imputed
PH, MVN−EM
PH, Mean
PH, RF
SPM, No−imputed
SPM, MVN−EM
SPM, Mean
SPM, RF
−log10(p)
0 1 2 3 4 5 6
1 5 9 15 20
Chromosome
